# Gestational diabetes in mice induces hematopoietic memory that affects the long-term health of the offspring

**DOI:** 10.1172/JCI169730

**Published:** 2024-01-16

**Authors:** Vinothini Govindarajah, Masahide Sakabe, Samantha Good, Michael Solomon, Ashok Arasu, Nong Chen, Xuan Zhang, H. Leighton Grimes, Ady Kendler, Mei Xin, Damien Reynaud

**Affiliations:** 1 Division of Experimental Hematology and Cancer Biology and; 2 Division of Immunobiology, Cincinnati Children’s Hospital Medical Center (CCHMC), Cincinnati, Ohio, USA.; 3 Department of Pediatrics and; 4 Department of Pathology, University of Cincinnati College of Medicine, Cincinnati, Ohio, USA.

**Keywords:** Hematology, Inflammation, Cardiovascular disease, Diabetes, Hematopoietic stem cells

## Abstract

Gestational diabetes is a common medical complication of pregnancy that is associated with adverse perinatal outcomes and an increased risk of metabolic diseases and atherosclerosis in adult offspring. The mechanisms responsible for this delayed pathological transmission remain unknown. In mouse models, we found that the development of atherosclerosis in adult offspring born to diabetic pregnancy can be in part linked to hematopoietic alterations. Although they do not show any gross metabolic disruptions, the adult offspring maintain hematopoietic features associated with diabetes, indicating the acquisition of a lasting diabetic hematopoietic memory. We show that the induction of this hematopoietic memory during gestation relies on the activity of the advanced glycation end product receptor (AGER) and the nucleotide binding and oligomerization domain-like receptor family pyrin domain-containing 3 (NLRP3) inflammasome, which lead to increased placental inflammation. In adult offspring, we find that this memory is associated with DNA methyltransferase 1 (DNMT1) upregulation and epigenetic changes in hematopoietic progenitors. Together, our results demonstrate that the hematopoietic system can acquire a lasting memory of gestational diabetes and that this memory constitutes a pathway connecting gestational health to adult pathologies.

## Abstract 2

Adult offspring born from diabetic pregnancy acquire a lasting hematopoietic memory that contributes to long-term adult pathology.

## Graphical abstract

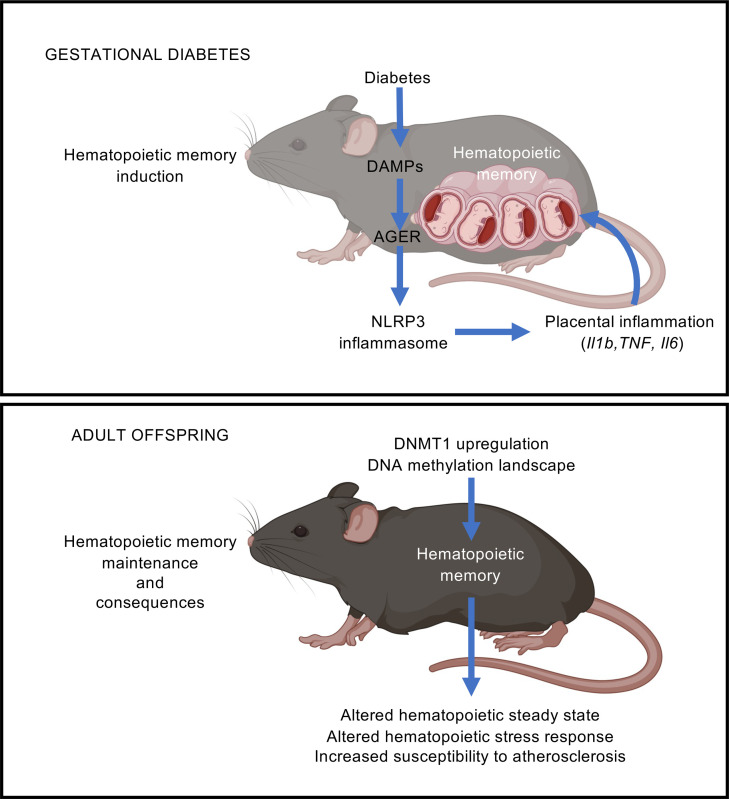

## Introduction

One in six pregnancies is affected by some form of gestational diabetes (GD), a prevalence that is steadily rising in the context of the worldwide epidemics of obesity and early diabetic onset (1). Of these cases, 80% are classified as gestational diabetes mellitus (GDM), which present an episode of glucose intolerance associated with the metabolic disruptions occurring during the second or third trimester of the pregnancy. The remaining cases arise earlier in pregnancy and are referred to as diabetes in pregnancy. They include women with prediabetes who become diabetic during pregnancy or women with overt type 1 or type 2 diabetes who have difficulty regulating their glycemia during pregnancy. When untreated, this condition has direct short-term consequences on pregnancy, with poor maternal and offspring outcomes, including increased risk of retinopathy, nephropathy, and preeclampsia for the mother and congenital cardiac malformation, macrosomia, and jaundice for the newborn (2, 3). Early diagnosis and advances in maternal glucose control have greatly mitigated the perinatal consequences of GD for the mothers and the offspring (2–4). However, these advances have only marginally affected its long-term consequences. Exposure to hyperglycemia in utero remains associated with long-term morbidities in adult offspring, including increased risk of metabolic diseases, atherosclerosis, and cardiovascular diseases (5–9). The mechanisms driving this pathological transmission across generations remain unknown.

Here we establish two reliable genetic and pharmacological mouse models that emulate the adverse perinatal and long-term consequences associated with GD in human. In these models, we reveal the existence of a lasting hematopoietic memory associated with GD that contributes to the increased susceptibility to atherosclerosis of the adult offspring. Mechanistically, we show that the acquisition of the hematopoietic memory during diabetic gestation relies on the activity of the advanced glycation end product receptor (AGER) pattern recognition receptor and the nucleotide binding and oligomerization domain-like receptor family pyrin domain-containing 3 (NLRP3) inflammasome to promote placental inflammation. Finally, we show that this memory is associated with epigenetic changes in hematopoietic stem and progenitor cells (HSPCs) and progenitor cells. Together, our results highlight important pathways that connect maternal and fetal health to adult pathologies. Notably, they demonstrate that the hematopoietic system can acquire a lasting memory of gestational events and that transgenerational hematopoietic memory could be an important “effector” of pathologies in adulthood.

## Results

### Two independent genetic and pharmacological models of diabetes in pregnancy.

**Figure 1:**
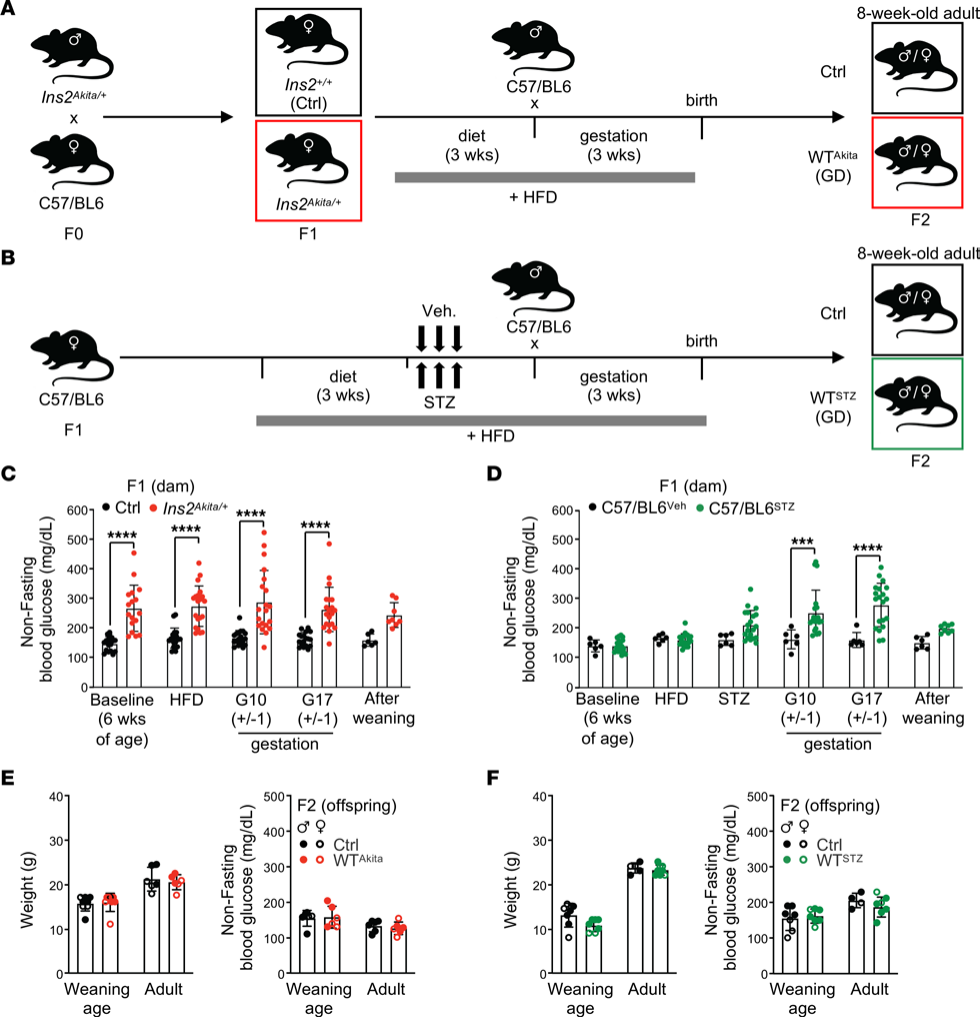
Modeling diabetes during pregnancy. (**A** and **B**) Breeding and treatment schematic used to generate genetic (**A**) and pharmacological (**B**) mouse models of GD. (**C** and **D**) Dam nonfasting glycemia before, during, and after pregnancy in genetic (**C**) and pharmacological (**D**) mouse models (**C**: *n* = 17–20/group, **D**: *n* = 6–20/group, *n* = 6–8/group at postweaning age). (**E** and **F**) Weaning-age (4 weeks old) and adult-age (8 weeks old) body weight (left panels) and nonfasting glycemia (right panels) of offspring born to diabetic pregnancy in the genetic (**E**) and pharmacological (**F**) mouse models (*n* = 5–8/group). Data are represented as means ± SD. Two-way ANOVA with Šidák’s post hoc test. ***P ≤ 0.0005; ****P ≤ 0.0001.

We conducted our studies in 2 independent mouse models of diabetes in pregnancy. First, we used a mouse genetic model of type 1 diabetes (*Ins2^Akita/J^*) under a rigorous breeding scheme (Figure 1A). C57BL/6-backcrossed *Ins2^Akita/J^* mice carry a spontaneous mutation in the *Ins2* gene that induces the toxic folding of the insulin protein, leading to reduced β cell mass and impaired insulin secretion (10). Mice heterozygous for the *Ins2^Akita^* mutation displayed normal weight and a diabetic profile with hyperglycemia, but not hyperinsulinemia. The colony was maintained by crossing *Ins2^Akita/+^* males to C57BL/6 females to limit intergenerational GD and potential germline alterations. To reinforce the hyperglycemia phenotype in females, F1 heterozygote *Ins2^Akita/+^* and WT littermate females were fed with high-fat diet (HFD) (60 kcal% fat) for 3 weeks before breeding with C57BL/6 males (Figure 1A). In this condition, *Ins2^Akita/+^* dams displayed high nonfasting blood-glucose levels during gestation (measured at gestational days G10±1 and G17±1) (Figure 1C). To independently validate our results, we established a pharmacological C57BL/6 mouse model, based on a 2-hit approach that combines HFD with 3 injections of streptozotocin (STZ) (60 mg/kg/d) to target maternal pancreatic β cells and induce diabetes (Figure 1B) (11). STZ administration was completed 1 week prior to mating, precluding any direct impact of STZ on the offspring. In this model, dams developed hyperglycemia during gestation with a late-stage peak at G17±1 (Figure 1D). Consistent with human pathology, both animal models showed an increase in perinatal adverse events (for ~15% of the dams), including cases of stalled labor, dystocia, and pup mortality (data not shown) (2). Offspring (F2) were maintained under regular 13 kcal% fat diet from birth into adulthood and analyzed as adults at 8 to 12 weeks of age. Analyses were performed on WT offspring that did not carry the diabetogenic mutation (denoted hereafter as WT^Akita^) or were not directly exposed to STZ treatment (denoted hereafter as WT^STZ^). GD offspring were compared with control (Ctrl) mice born to normal pregnancy generated through a similar breeding scheme. As expected, GD offspring showed no gross metabolic abnormalities at weaning (4 weeks) and adulthood (8 weeks) when assessed by (a) body weight, (b) nonfasting or fasting blood glucose, and (c) glucose-tolerance test (GTT) or insulin-tolerance test (ITT) (Figure 1, E and F, and Supplemental Figure 1, A and B; supplemental material available online with this article; https://doi.org/10.1172/JCI169730DS1). Together, our results describe 2 independent GD mouse models with gestational hyperglycemia and perinatal adverse features that recapitulate human pathology. They show that offspring born to these models of diabetic pregnancy do not present major metabolic abnormalities or glycemic alterations when analyzed during early adulthood.

### Diabetic pregnancy promotes atherosclerosis development in adult offspring.

**Figure 2:**
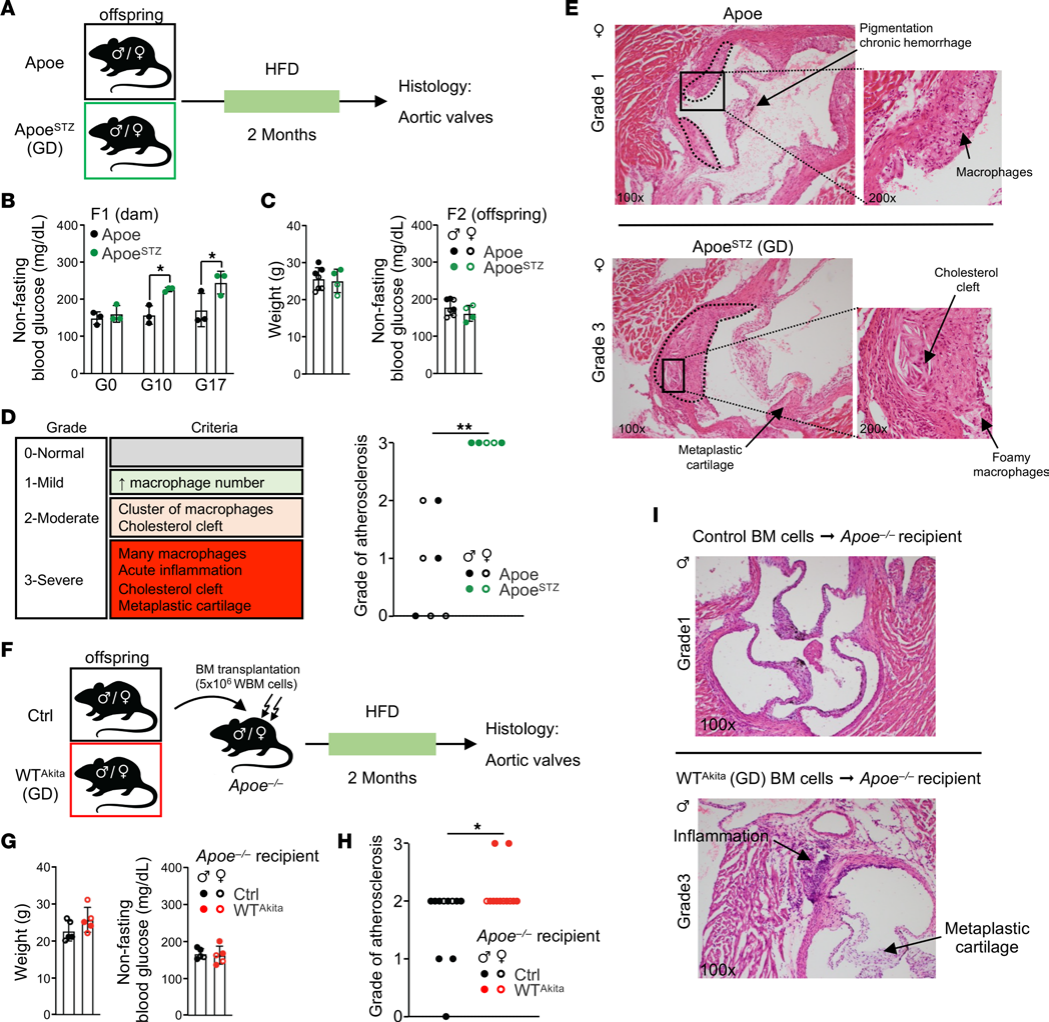
GD promotes atherosclerosis development in adult offspring. (**A**) Experimental schematic used to assess atherosclerosis development in *Apoe^–/–^* GD offspring. (**B**) STZ-treated *Apoe^–/–^* dam nonfasting glycemia before and during pregnancy (*n* = 3). (**C**) Adult body weight (left panel) and nonfasting glycemia (right panel) of *Apoe^–/–^* offspring born to diabetic pregnancy (*n* = 4–7). (**D**) Histological criteria and atherosclerosis severity score in adult Ctrl and GD *Apoe^–/–^* offspring (*n* = 7–5/group). (**E**) Example of histological analysis of H&E-stained aortic valve from *Apoe^–/–^* offspring born to diabetic pregnancy compared with Ctrl. Original magnification, ×100; insets, ×200. (**F**) Experimental schematic used to assess atherosclerosis in *Apoe^–/–^* recipients transplanted with BM isolated from adult Ctrl and GD *Apoe^–/–^* offspring. (**G**) Adult body weight (left panel) and nonfasting glycemia (right panel) of *Apoe^–/–^* recipient mice transplanted with BM cells isolated from Ctrl or GD offspring (*n* = 5). (**H**) Atherosclerosis severity in *Apoe^–/–^* recipient mice (*n* = 11–12/group). (**I**) Example of histological analysis of aortic valve from *Apoe^–/–^* recipient mice. Original magnification, ×100. Data are represented as means ± SD (**B**, **C**, and **G**). Two-way ANOVA with Šidák’s post hoc test (**B**); χ^2^ test for trend (**D** and **H**). *P ≤ 0.05; **P ≤ 0.01.

In human populations, GD has been associated with early onset of atherosclerosis development in offspring (8, 9). To assess whether the mouse models mimic human pathology, we induced GD in atherosclerosis-prone *Apoe*-KO mice using the STZ protocol and challenged the offspring with HFD to favor atherosclerosis development (Figure 2, A and B). As described in WT mice, adult GD *Apoe^–/–^* offspring did not display any changes in body weight or gross glycemic alterations (Figure 2C). However, aortic valve histological examinations showed accelerated atherosclerosis development in GD offspring compared with Ctrls (Figure 2D). Notably, we observed increased inflammation, lipid deposition, and cartilaginous metaplasia in the aortic valves (Figure 2E and Supplemental Figure 2, A and B). We then used hematopoietic transplantation to assess the contribution of the hematopoietic system to the atherosclerotic phenotype. Saturating amounts of BM cells (5 × 10^6^) isolated from Ctrl or GD WT^Akita^ offspring were transplanted into *Apoe^–/–^* irradiated recipient mice that were subsequently challenged with HFD (Figure 2F). Transplanted mice showed no overt glycemic alterations (Figure 2G). The severity of the atherosclerotic features was reduced in *Apoe^–/–^* recipient mice, consistent with previous reports (12, 13). However, even in this context, recipient mice transplanted with GD offspring BM showed an increase in aortic valve atherosclerotic lesions (Figure 2, H and I, and Supplemental Figure 2C). These lesions in the *Apoe^–/–^* recipients were associated with exacerbated monocytosis and accumulation of aortic myeloid cells (Supplemental Figure 2, D and E) (14, 15). Together, these results validate the use of the 2 GD mouse models to mimic the pathological conditions described in human cohorts born to diabetic pregnancy. They also suggest that the hematopoietic system contributes, at least to some extent, to atherosclerosis development in adult GD offspring.

### Long-term alterations of the steady-state hematopoiesis in offspring born to diabetic pregnancy.

**Figure 3:**
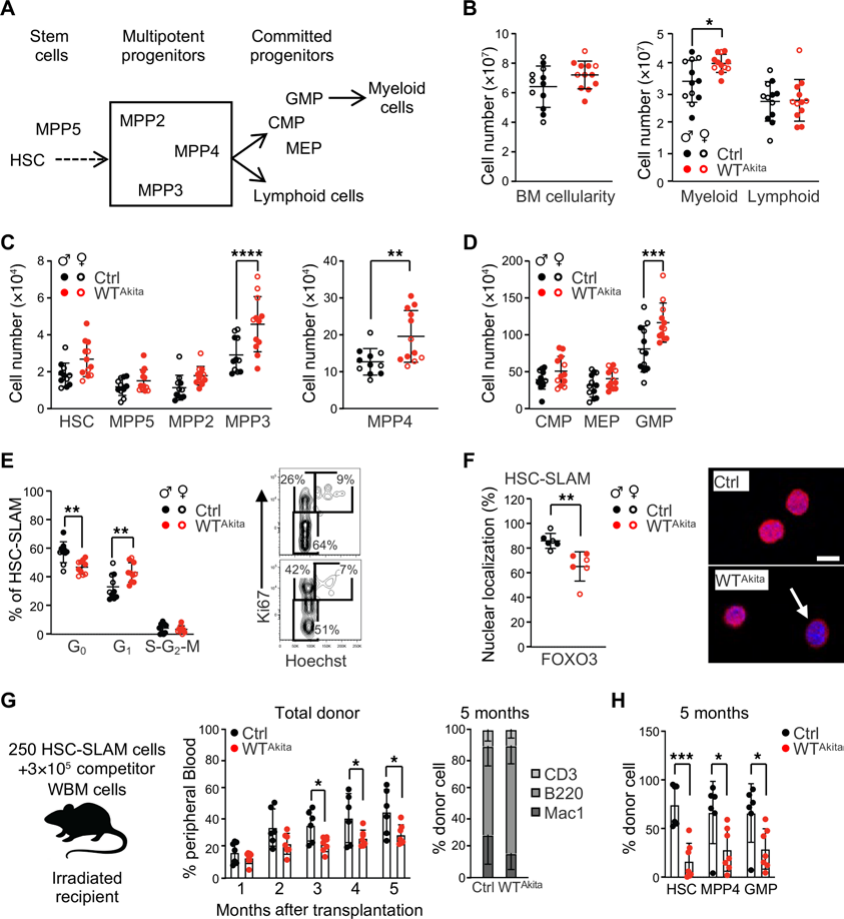
Offspring born to diabetic pregnancy display altered steady-state hematopoiesis. (**A**) Schematic of the murine hematopoietic hierarchy. (**B**) BM cellularity and absolute numbers of BM myeloid/lymphoid cells in adult WT^Akita^ offspring (*n* = 12). (**C** and **D**) Absolute numbers of HSPC populations in the BM of adult Ctrl and WT^Akita^ offspring (*n* = 11–12). CMP, common myeloid progenitor (Lineage^–^c-Kit^+^Sca-1^–^CD34^+^FcγR^–^); MEP, megakaryocyte/erythroid progenitor (Lineage^–^c-Kit^+^Sca-1^–^CD34^–^FcγR^–^). (**E**) Percentage of HSC distribution in cell-cycle phases in adult Ctrl and WT^Akita^ offspring. Right panel shows representative FACS plots for Ki67/ Hoechst 33342 staining (*n* = 10). (**F**) Percentage of HSCs isolated from Ctrl and WT^Akita^ offspring that present FOXO3 nuclear localization at steady state (*n* = 6 with 50 individual cells analyzed for each). Right panel shows representative images of FOXO3 immunofluorescence analysis. Scale bar: 10 μm. (**G** and **H**) Competitive hematopoietic reconstitution assay for HSCs isolated from Ctrl (*n* = 6) and WT^Akita^ (*n* = 7) offspring from 1 experiment: PB chimerism over time (**G**) and BM chimerism for HSC subsets, 20 weeks after transplantation (**H**). Data are represented as means ± SD. Two-way ANOVA with Šidák’s post hoc test (**B**, **C**, **D**, **G**, and **H**) or with Tukey’s post hoc test (**E**); unpaired 2-tailed Student’s *t* test (**F**). *P ≤ 0.05; **P ≤ 0.01; ***P ≤ 0.0005; ****P ≤ 0.0001.

We next performed a comprehensive analysis of the hematopoietic compartments present in the BM of 8-week-old WT^Akita^ mice born to diabetic pregnancy (Figure 3A) (16). While BM cellularity was not dramatically affected, we found that steady-state hematopoiesis was skewed toward the myeloid lineage (Figure 3B). We analyzed the immature Lineage^–^c-Kit^+^Sca-1^+^ (LSK) BM compartment subfractionated based on the expression of the marker Flt3, CD150, and CD48 (Figure 3C and Supplemental Figure 3A). GD did not affect the frequency and phenotype of the hematopoietic stem cells (HSCs) defined as LSK/Flt3^–^CD48^–^CD150^+^ (HSC-SLAM). In contrast, we observed an expansion of the short-term multipotent progenitors MPP3 and MPP4, defined as LSK/Flt3^–^CD48^+^CD150^–^ and LSK/Flt3^+^CD48^+^CD150^–^, respectively. This was associated with an expansion of the myeloid committed progenitors (granulocyte/macrophage progenitor [GMP]: Lineage^–^c-Kit^+^Sca-1^–^CD34^+^FcγR^+^) (Figure 3D). Functionally, a decrease in the quiescence of the HSC compartment was detectable by intracellular Hoechst 33342/Ki67 staining (Figure 3E). HSC activation was further confirmed by reduced nuclear localization of FOXO3, a key regulator of HSC quiescence (17) (Figure 3F). This activated phenotype was associated with a loss of HSC fitness in competitive transplantation assay (Figure 3, G and H) (18). Similar BM myeloid skewing was observed in the pharmacological STZ model with an expansion of the MPP4 and GMP compartments (Supplemental Figure 3, B–D). Although we did not observe a major alteration of the HSC quiescence at steady state, competitive transplantation assay revealed a similar loss of HSC fitness (Supplemental Figure 3, E–H).

Together, these results suggest a long-lasting effect of GD on the offspring hematopoietic system that persists into adulthood. We observed marks of activation of the HSC compartment and an expansion of key hematopoietic myeloid progenitors (MPP and GMP). Although these alterations did not lead to any overt hematological pathologies at the time of analysis, they signal the persistence of a latent dysregulated hematopoietic state in offspring born to diabetic pregnancy.

### GD offspring display altered hematopoietic response to inflammatory cues.

**Figure 4:**
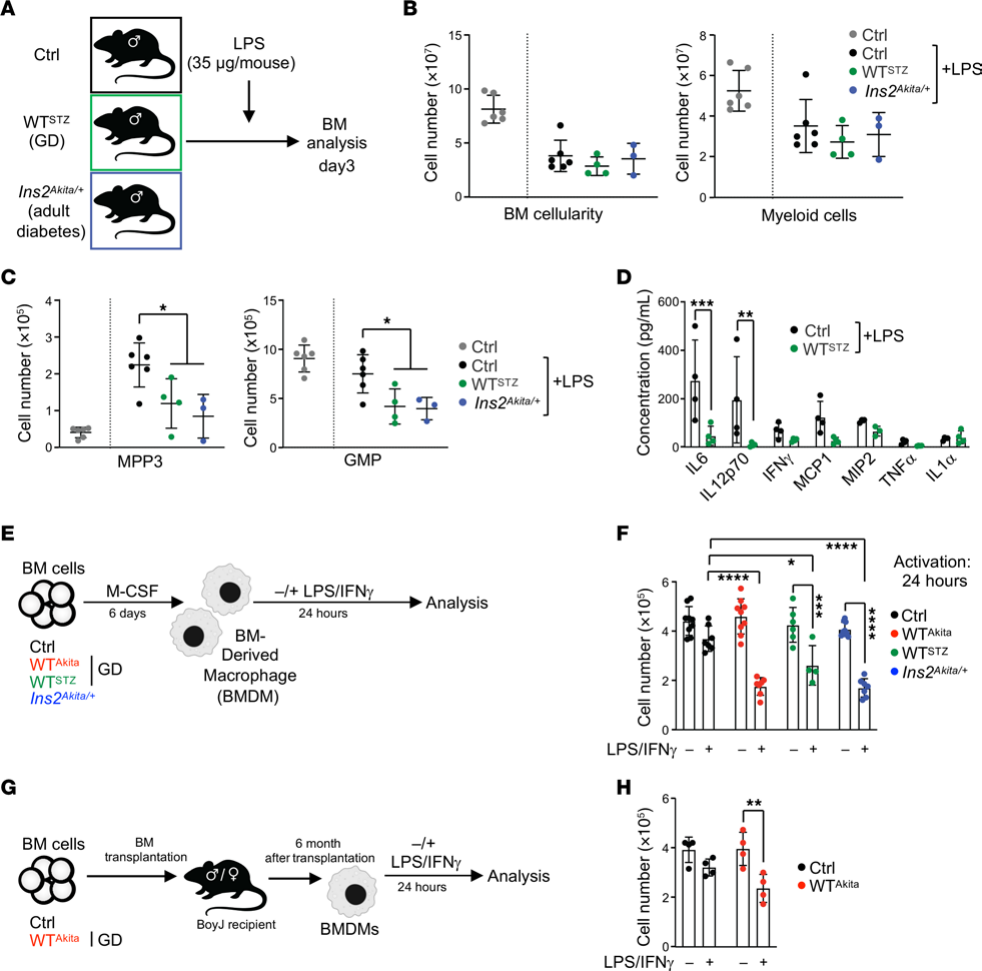
GD altered the inflammatory hematopoietic response in offspring. (**A**) Schematic of the experimental design for the in vivo LPS inflammatory challenge. (**B**) BM cellularity and absolute numbers of BM myeloid cells 3 days after LPS treatment. (**C**) Absolute numbers of BM MPP3 and GMP cells 3 days after LPS treatment (*n* = 3–6/group). (**D**) Inflammatory cytokine concentration in the mouse serum 3 days after LPS treatment (*n* = 4/group). (**E**) Schematic of experimental design to generate and activate BMDMs. (**F**) Absolute numbers of BMDMs in culture 24 hours after treatment with PBS or LPS/IFN-γ (*n* = 4–9/group). (**G**) Schematic of experimental design describing BMDM generation from transplanted mice. (**H**) Following transplantation, absolute numbers of BMDMs in culture 24 hours after treatment with PBS or LPS/IFN-γ (*n* = 4/group). Data are represented as means ± SD. One-way ANOVA with Tukey’s post hoc test (**B** and **C**) or 2-way ANOVA with Šidák’s post hoc test (**D**, **F** and **G**). *P ≤ 0.05; **P ≤ 0.01; ***P ≤ 0.0005; ****P ≤ 0.0001.

We assessed the functional effect of GD on the ability of the offspring hematopoietic system to respond to inflammatory challenge (Figure 4A). We used LPS to mimic bacterial infection in Ctrl and GD WT^STZ^ offspring and in adult diabetic *Ins2^Akita/+^* males (nonfasting blood glucose: 390.7 ± 57.7 mg/dL, *n* = 10). As expected, LPS treatment led in all conditions to a decrease of the BM cellularity and a reduction of the number of BM myeloid cells (Figure 4B). In contrast, we observed an altered hematopoietic stress response in WT^STZ^ GD offspring and diabetic *Ins2^Akita/+^* mice, phenotypically characterized by limited MPP3 expansion and a slow recovery of the GMP compartment (Figure 4C). This phenotype in GD offspring was associated with a reduced inflammatory cytokine response, particularly for IL-6, IL-12p70, and, to a lesser extent, IFN-γ, TNF-α, MCP1, and MIP2 (Figure 4D). It is noteworthy that similar alterations were found in a model of viral infection using polyinosinic:polycytidylic acid (pIC), suggesting that these functional characteristics are not linked to a specific inflammatory pathway (Supplemental Figure 4, A–D). These results indicate that GD leads to lasting disruption of the hematopoietic stress response in the offspring. Although GD offspring do not display any gross metabolic defects, we observed that they mimic the hematopoietic features found in diabetic mouse models. Thus, these results suggest the existence of a long-term functional glycemic memory in adult offspring born to diabetic pregnancy.

To confirm these observations, we generated BM-derived macrophages (BMDMs) from adult Ctrl, GD WT^Akita^, and WT^STZ^ offspring, along with diabetic *Ins2^Akita/+^* mice (Figure 4E). We did not observe any qualitative or quantitative defects in BMDM generation based on cell number and immunophenotype (data not shown). As expected, all BMDMs acquired an inflammatory phenotype upon treatment with LPS (10 ng/mL) and IFN-γ (10 ng/mL), as assessed by the acquisition of the CD86 marker and the expression of inflammatory cytokines such as *Il6*, *Il1a*, and *Tnf* (Supplemental Figure 4E and data not shown). However, we found that BMDMs generated from WT^Akita^ and WT^STZ^ GD offspring were reduced in number after activation compared with Ctrl (Figure 4F). This loss of cellularity was detectable as early as 3 hours after activation (Supplemental Figure 4F). Consistent with the maintenance of a functional glycemic memory, this property of BMDMs generated from GD offspring mimicked the behavior of BMDMs derived from diabetic *Ins2^Akita/+^* mice. Importantly, this property was maintained when GD offspring BM cells were transplanted into normal congenic mice (Figure 4G). Thus, BMDMs generated from recipient mice 6 months after BM transplantation maintained a heightened sensitivity to inflammation (Figure 4H). Together, these data show that BMDMs generated from GD offspring and diabetic mice share functional properties that are distinct from those of Ctrl BMDMs. Results in the BM transplantation setting demonstrate that the diabetic memory generated in GD offspring is an intrinsic hematopoietic property supported by alterations in the HSC compartment.

### Sterile inflammation contributes to the induction of the GD hematopoietic memory during pregnancy.

**Figure 5:**
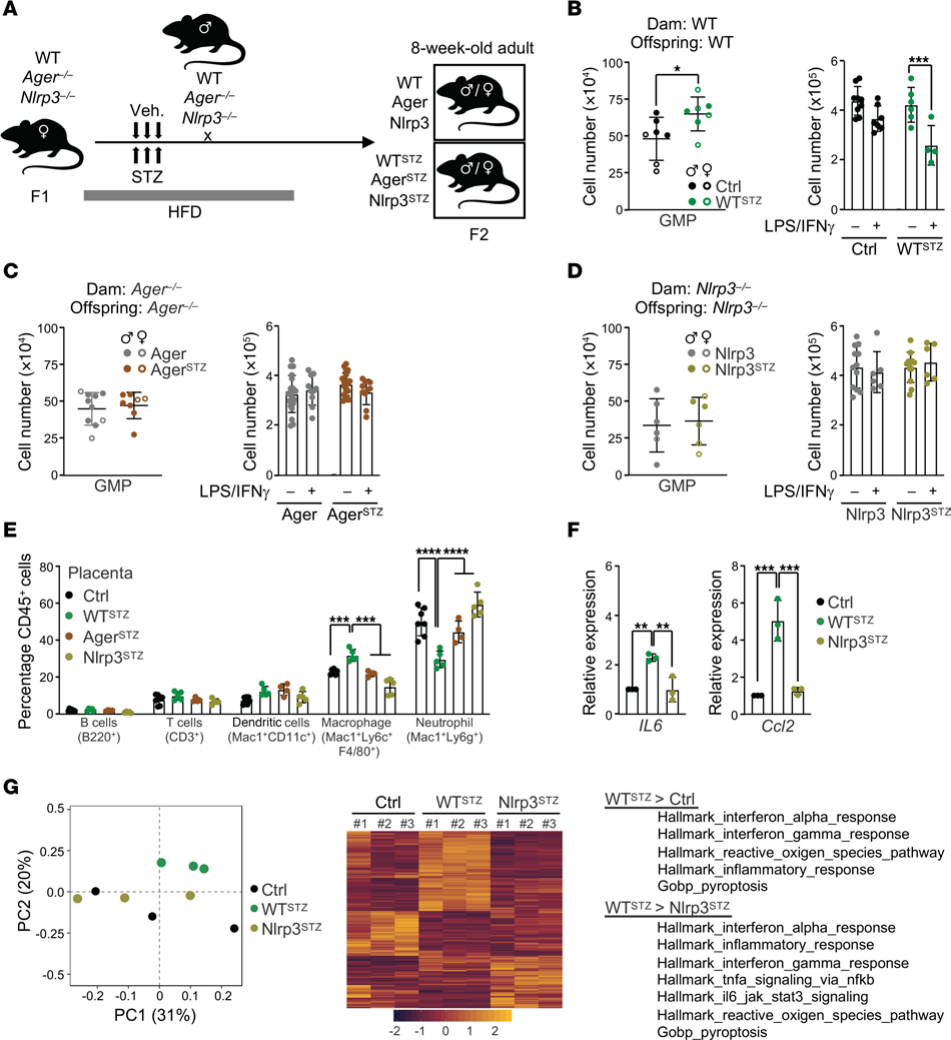
AGER and NLRP3 function are necessary for the induction of the GD hematopoietic memory in offspring. (**A**) Schematic of the experimental design. (**B**–**D**) Hematopoietic readout for offspring born to normal or diabetic pregnancy: absolute numbers of BM GMP cells (left graphs) and absolute numbers of BMDMs in culture 24 hours after treatment with PBS or LPS/IFN-γ (right graphs) in WT (**B**) (data also presented in Supplemental Figure 3D and Figure 4F), *Ager^–/–^* (**C**), and *Nlrp3^–/–^* backgrounds (**D**). (**C**: *n* = 8–10 for GMP; *n* = 10–20 for BMDM; **D**: *n* = 6 for GMP, *n* = 6–12 for BMDM). (**E**) Immune cell composition in placenta: percentages in CD45^+^ cells (*n* = 4–7/group). (**F**) RT-PCR analysis showing the expression of the *Il6* and *Ccl2* inflammatory cytokine genes. Results are expressed as fold change relative to Ctrls, set at 1 (*n* = 3). (**G**) RNA-Seq analysis on placental CD45^+^ cells isolated at G17 from Ctrl, WT^STZ^, and Nlrp3^STZ^ dams (*n* = 3/group). Principal component analysis (PCA) of RNA-Seq data (left panel). Differential gene signature: heatmap using differentially expressed genes (DEGs) with log_2_FC> |0.58| and FDR < 0.05 in all comparisons (central panel). GSEA of genes differentially expressed in WT^STZ^ cells compared with Ctrl and Nlrp3^STZ^ conditions (right panel). Data are represented as means ± SD. Unpaired 2-tailed Student’s *t* tests (**B**–**D**, left graphs); 1-way ANOVA with Tukey’s post hoc test (**F**) or 2-way ANOVA with Šidák’s post hoc test (**B**–**D**, right graphs; **E**). *P ≤ 0.05; **P ≤ 0.01; ***P ≤ 0.0005; ****P ≤ 0.0001.

We hypothesized that damage-associated molecular patterns (DAMPs) linked to hyperglycemia could contribute to the in utero induction of the GD hematopoietic memory (19). AGER is a receptor for several metabolic stress signals, such as advanced glycation end products (AGEs), HMGB1, and S100 proteins (20). Previous reports have demonstrated the role of AGER in GD-associated fetal alterations (21, 22). We used a loss-of-function approach to determine the contribution of AGER to the development of a GD hematopoietic memory. We treated *Ager*-deficient (*Ager^–/–^*) dams with STZ to generate GD mutant offspring (Ager^STZ^) (Figure 5A). Mutant dams did not show any alterations of the diabetic phenotype during pregnancy compared with their WT counterparts (Supplemental Figure 5A). Adult GD offspring were evaluated by BM phenotypic analysis at steady state and functional BMDM assessment, two defining criteria of GD hematopoietic memory in WT mice (Figure 5B). Based on these readouts, we observed that the disruption of the AGER pathway blocks the acquisition of the GD hematopoietic memory in offspring (Figure 5C). Targeted gene invalidation in the dam (by crossing male WT with female *Ager^–/–^*) or in the fetus (by crossing male *Ager^–/–^* with female *Ager^+/–^* and selecting *Ager^–/–^* offspring) demonstrate that this pathway is specifically required in the mother for the induction of the GD hematopoietic memory in the offspring (Supplemental Figure 5, B and C). These results show that the maternal AGER pathway is a primary inducer of the diabetic hematopoietic memory and suggest the existence of secondary signals that affect the hematopoiesis of the offspring.

We hypothesized that sterile inflammation could be central to these secondary signals. We tested the NLRP3 inflammasome, which is a known regulator of sterile inflammation and which has been associated with GD and pregnancy complications (23, 24). We assessed NLRP3 as previously described for AGER (Figure 5A). Global NLRP3 targeting did not affect the dam GD (Supplemental Figure 5D), but did prevent the acquisition of the GD hematopoietic memory in offspring (Nlrp3^STZ^) (Figure 5D). Unlike AGER, NLRP3 was required in both the dam and the fetus (Supplemental Figure 5, E and F). Furthermore, we found that the GD hematopoietic memory correlates with placental inflammation. We observed an expansion of the macrophage population in placenta of WT^STZ^ offspring that develop a hematopoietic memory, but not in Ager^STZ^ and Nlpr3^STZ^ offspring (Figure 5E). As expected, accumulation of placental macrophage was associated with expression of inflammatory cytokine genes, such as *Il6*, *Ccl2*, *Il1b*, and *Tnf*. (Figure 5F and Supplemental Figure 5G). These results were strengthened by RNA-Seq analyses performed on CD45^+^ cells isolated from placenta, which confirmed the link between GD and placental inflammation and its dependence on NLRP3 (Figure 5G, Supplemental Figure 5H, and Supplemental Table 1). Together, these results show that induction of the GD hematopoietic memory in offspring requires the AGER/NLRP3 pathways and is associated with sterile placental inflammation.

### DNA methylation contributes to the maintenance of the GD hematopoietic memory during adulthood.

**Figure 6:**
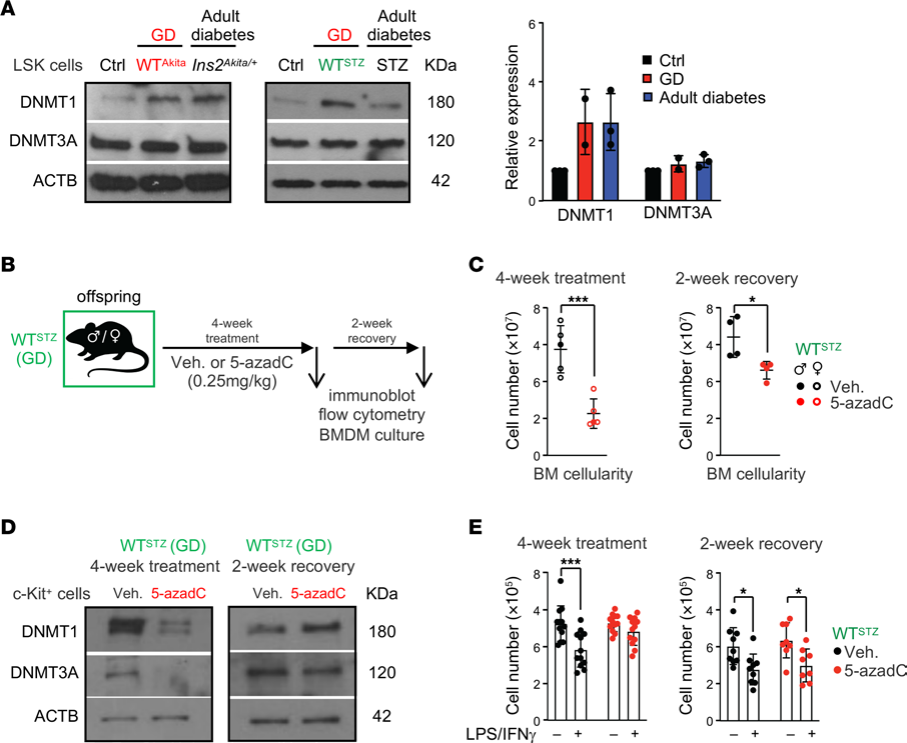
DNA methylation contributes to the maintenance of GD hematopoietic memory in adult. (**A**) Immunoblot analysis of DNMT1 and DNMT3A in LSK cells isolated from Ctrl, GD offspring, (WT^Akita^ and WT^STZ^), and adult mice with full-blown diabetes triggered by the *Ins2^Akita^* mutation or STZ treatment. (**B**) Schematic of experimental design for in vivo treatment with vehicle or 5-azadC. (**C**) BM cellularity in WT^STZ^ offspring directly after 4 weeks of 5-azadC treatment (*n* = 5/group) or a 2-week recovery period after treatment (*n* = 4/group). (**D**) Immunoblot analysis of DNMT1 and DNMT3A in c-Kit^+^ cells isolated from WT^STZ^ after 5-azadC treatment or recovery period. (**E**) Absolute numbers of BMDMs in culture 24 hours after treatment with PBS or LPS/IFN-γ for 5-azadC–treated WT^STZ^ offspring after treatment (*n* = 12) or recovery period (*n* = 8). Data are represented as means ± SD. Unpaired 2-tailed Student’s *t* tests (**C**) and 2-way ANOVA with Šidák’s post hoc test (**E**). *P ≤ 0.05; ***P ≤ 0.0005.

Next, we investigated the mechanisms that support the persistence of the GD hematopoietic memory in adult offspring. Diabetes has been associated with epigenetic changes (25). Particularly, hematopoietic alterations in diabetic mice have been linked to alterations in the DNA methylation landscape and increased expression of DNA methyltransferase 1 (Dnmt1) (26). In LSK cells, we confirmed a specific increase of DNMT1 but not DNMT3a protein in adult diabetic *Ins2^Akita/+^* and STZ-treated mice (Figure 6A). Interestingly, we found that LSK cells from GD adult offspring show a similar increase of DNMT1 protein, even as the models do not display overt hyperglycemia. Using reduced representation bisulfite sequencing (RRBS) analysis, we found that DNMT1 upregulation correlates with methylome alterations in HSPCs of adult GD offspring. It particularly affected genes that respond to reactive oxygen/nitrogen species, which are important features of diabetes (27), and gene pathways previously found differentially methylated in cord blood cells from diabetic pregnancy (e.g., cell-cell adhesion, MAPK signaling, cytosolic transport) (28) (Supplemental Figure 6A and data not shown). These results were reinforced by transposase-accessible chromatin sequencing (ATAC-Seq) assay performed in LSK cells isolated from adult Ctrl and GD offspring (Supplemental Figure 6, B–D). Despite some degree of variability between replicates, differential analysis of the accessible sites showed differences in the chromatin structure in Ctrl and GD LSK compartments. Particularly, we observed a reduced accessibility in GD offspring that affects genes involved in metabolism, oxidative stress, and inflammation pathways. Although limited in scope, these analyses are consistent with the idea of epigenetic alterations in GD offspring. To assess the contribution of DNA methylation to the GD hematopoietic memory, we used a low dose of the DNA methyltransferase inhibitor 5-aza-2′-deoxycytidine (5-azadC) to reset the methylome profile in GD offspring (29, 30) (Figure 6B). GD WT^STZ^ offspring were analyzed immediately after treatment or following a 2-week recovery period. We observed that 5-azadC treatment affects BM cellularity without dramatically disturbing the hematopoietic hierarchy assessed by flow cytometry (Figure 6C and Supplemental Figure 6E). Hematopoietic parameters were normalized after the end of treatment (30). As expected, DNMT1 and DNMT3a protein levels in HSPCs were reduced by 5-azadC treatment, but were restored following treatment cessation (Figure 6D). By assessing the BMDM function, we found that hypomethylating treatment limits the loss of BMDM cellularity following inflammatory activation, consistent with a loss of the GD hematopoietic memory (Figure 6E). However, we found that this effect was temporary, as the BMDM phenotype remerges after treatment cessation (Figure 6E). Consistent with the idea of a GD hematopoietic memory, these results show that offspring born to diabetic pregnancy maintain into adulthood high expression of DNMT1, a molecular feature associated with overt diabetes. In addition, these results suggest that upregulation of DNMT1, and the associated changes in DNA methylation, is one of the factors supporting the GD memory in the hematopoietic system.

## Discussion

Our results demonstrate the existence of a hematopoietic memory associated with GD. It is tempting to speculate that this phenomenon is related to the hematopoietic memory recently established in the context of an immune response (31). Referred to as trained immunity, this concept proposes that the hematopoietic system not only directly responds to inflammatory signals but also can “remember” the inflammatory events, therefore heightening or dampening the response to secondary challenges (32, 33). This property, uncovered in short-lived innate immune cells, such as monocytes and macrophages, was expanded to hematopoietic progenitor cells (34, 35). Its persistence through BM transplantation suggests that it originates in HSCs (36, 37). Consistent with this finding, our work indicates that the HSC compartment is altered by GD, as shown by the persistence of the GD phenotype for over an 8-week growth period, from the neonatal period to adulthood. In adult GD offspring, we found an increased HSC activation at steady state and a loss of fitness upon transplantation. We observed that the abnormal BMDM function in GD offspring could be maintained in the BM transplantation setting for over a 6-month period. These results confirm that the hematopoietic system, and particularly the HSC compartment, are reactive to organismal metabolic stresses (17, 18, 38). They are also consistent with recent findings showing that endogenous “sterile” signals associated with Western diet or hyperglycemia can induce a memory in innate immune cells and their precursors (39–41).

Mechanistically, our work describes a scenario in which GD-associated stress signals promote the induction of the hematopoietic memory through activation of the AGER pathway. While AGER expression is low in most cell types in healthy conditions, it is upregulated in several disease states, including diabetes (42). In adults, AGER signaling has been shown to promote myelopoiesis in a hyperglycemic environment (43). AGER and its ligands S100A8/A9 also play a dominant role in myelopoiesis in the context of intermittent glycemic fluctuation (44). During pregnancy, AGER contributes to preeclampsia, preterm birth, and different congenital malformations associated with diabetic pregnancy (21, 22). Our data indicate that the AGER pathway is an essential upstream inducer of the GD hematopoietic memory. AGER belongs to a class of pattern-recognition receptors that recognize broad common features. We notice that Toll-like receptors (TLR4/TLR2), which share common ligands and signaling pathways with AGER, were not able to compensate for AGER deficiency (45, 46). This specific requirement of AGER may rely on specific ligands, which remain to be determined. It may also be related to a specific pattern of expression. Differential dam/offspring loss-of-function experiments demonstrate that maternal AGER is the contributor of this effect while fetal AGER is dispensable. This maternal specificity reveals the existence of secondary signals able to promote hematopoietic alterations in offspring. Consistent with this idea, we found that the maternal and fetal NLPR3 inflammasome are required for the induction of the GD hematopoietic memory. Although our results do not formally link AGER and NLRP3 activity, our work indicates that these signaling pathways affect the level of placental inflammation. We envision that the developmental window conducive to the acquisition of the diabetic memory occurs in late gestation during the HSC transition from a fetal to an adult identity, a stage that has been shown to be sensitive to inflammatory signals (47). We speculate that some of the mechanisms revealed in our study could also contribute to the persistent immune alterations recently described in the context of infection occurring during pregnancy (48). Together, our results are consistent with the well-established role of AGER and NLRP3 in controlling the physiological and pathological inflammatory processes of pregnancy (23, 49). They are also in line with the hematopoietic impact of these signaling pathways in situations of metabolic stress triggered by either oxidized low-density lipoprotein or hyperglycemia (40, 41, 43). Finally, our results highlight that AGER-dependent “sterile” inflammation processes occurring during pregnancy are not only key determinants of the immediate pregnancy outcome, but also affect the long-term health of the offspring (50).

Epigenetic alteration acquired in utero may constitute the link between GD and its effects on the health of the adult offspring (51). Human studies using placenta, umbilical cord, or adult peripheral blood as well as skeletal muscle and adipose tissue suggest persisting epigenetic alterations in the offspring, including DNA methylation, histone modifications, and miRNA expression (28, 52–55). However, these studies in adults were limited by the intrinsic diversity of the human populations, the multiple confounding environmental factors affecting the epigenome, and the difficulty in experimentally assessing the clinical relevance of these alterations. Mouse models bypass these limitations and therefore could be important tools for studying transgenerational transmission (56, 57). While our results in mouse models correlate DNMT1 upregulation with the persistence of a GD hematopoietic memory during adulthood, the full impact of DNMT1 overexpression on the DNA methylation landscape and chromatin structure in HSPCs remains to be elucidated. We found that treatment with a DNA methyltransferase inhibitor disrupts the maintenance of the hematopoietic memory. However, we notice that this pharmacological approach is temporary, as GD hematopoietic memory is rapidly restored after treatment. This may be linked to the limited ability of the pharmacological approach to effectively target and reprogram the immature HSCs that sustain this phenotype. Alternatively, this may suggest the existence of other epigenetic mechanisms able to restore increased DNMT1 expression and contribute to the maintenance of GD hematopoietic memory. As DNMT1 expression emerges as a marker of the impact of prenatal and adult glycemia on the hematopoietic system, a deeper analysis of the regulation of the *Dnmt1* locus in diabetes could improve our understanding of the mechanisms controlling the maintenance of the hematopoietic memory in offspring born to diabetic pregnancy (26).

Our work shows that GD hematopoietic memory is associated with the alteration of the hematopoietic response to acute inflammatory stress. In adult GD offspring, we observed a decrease in production of inflammatory cytokine in response to LPS. Similarly, BMDMs derived from GD offspring displayed an increased susceptibility to inflammatory signals that led to a reduced cellularity in culture. This inflammatory dampening contrasts with the apparent contribution of the GD hematopoietic memory to the development of atherosclerosis in offspring. The connection between these seemingly contradictory phenotypes remains to be established. Previous studies showed that myeloid cells isolated from diabetic patients and BMDMs cultured in diabetic conditions display a heightened activation of the NLRP3 inflammasome (58, 59). Here, we show that the NLRP3 inflammasome is required for the acquisition and/or the manifestation of the hematopoietic GD memory in offspring. In this context, we speculate that the GD hematopoietic response to acute inflammatory stimulation could be linked to NLRP3 hyperactivation, leading to an inflammatory form of cell death known as pyroptosis (60). Interestingly, pyroptosis is a key promoter of the inflammatory phenotype fueling the initiation and the progression of atherosclerosis (61). We propose that, in chronic atherogenic conditions, heightened NLRP3 activation in hematopoietic cells may promote the atherosclerosis development associated with GD. Further studies are needed to fully evaluate the status of the NLRP3 pathway in GD offspring and test its contribution to the pathological consequences of GD hematopoietic memory.

This work exemplifies how prenatal health can have broad and lasting consequences on adult health. It particularly highlights the unappreciated contribution of the hematopoietic system in the transgenerational transmission of cardiovascular pathologies, such as atherosclerosis. By controlling the production of immune cells, the hematopoietic system is at the center of multiple pathological conditions and diseases. In this context, our work suggests that the induction and the lasting maintenance of a hematopoietic memory in offspring born to diabetic pregnancy alter their inflammatory stress responses and contribute to the development of chronic disease by creating vulnerability to lifestyle and environmental factors.

## Methods

### Mice.

Wild-type C57BL/6J (B6.SJL-Ptprca, strain 000664), Pepcb/BoyJ (strain 002014), *Ins2^Akita/J^* (strain 003548), *Ager^–/–^* (strain 032771), *Apoe^–/–^* (strain 002052), and *Nlrp3^–/–^* (strain 021302; gift from R. Marsh, CCHMC) mice were purchased from The Jackson Laboratory. Depending on the experimental design, mice were maintained on a Ctrl diet with 13 kcal% fat (5010; Lab Diets) or switched to HFD with 60 kcal% fat (D12492; Research Diet Inc.). In the pharmacological model, females were fasted for 6 hours before STZ treatment (60 mg/kg body weight). Animal breeding was performed over the weekend, leading to approximation of the gestational age of ±1 day.

### Reagents and resources.

A list of reagents and resources is presented in Supplemental Table 2.

### Blood glucose measurement.

Blood was sampled from the tail, and blood glucose was measured using an Accu-Chek Performa Glucometer. Nonfasting glucose was measured at fixed times between 10:00 am and 12:00 pm. For intraperitoneal ITT and oral GTT, adult offspring were fasted 6 and 12 hours, respectively, before receiving insulin (0.5 U/kg body weight) by intraperitoneal injection or glucose (2 g/kg body weight) per oral gavage. Blood glucose was measured before treatment to determine fasting glucose levels and every 15 minutes after insulin/glucose treatment.

### In vivo treatment.

BM from 8-week-old Ctrl and WT^STZ^ offspring along with diabetic *Ins2^Akita/+^* males was analyzed by flow cytometry 3 days after intraperitoneal treatment by LPS (35 μg/mouse) or pIC (10 μg/g body weight). 5-azadC intraperitoneal injection (0.25 mg/kg body weight) was performed 3 times a week (Monday, Wednesday, Friday) starting at 5 weeks of age.

### BM transplantation.

For competitive BM transplantation assay, 250 FACS-purified HSCs (HSC-SLAM) were mixed with 3 × 10^5^ unfractionated whole BM cells from Pepcb/BoyJ mice (CD45.1^+^) and retroorbitally injected into lethally irradiated CD45.1^+^ Pepcb/BoyJ recipient mice (11 Gy, delivered in split doses 3 hours apart). Hematopoietic reconstitution was monitored by flow cytometry on peripheral blood collected retroorbitally. HSPC donor chimerism was assessed in the BM by flow cytometry 20 weeks after transplantation. For noncompetitive transplantation, 5 × 10^6^ whole BM cells were retroorbitally transplanted into lethally irradiated CD45.1^+^ Pepcb/BoyJ or *Apoe^–/–^* recipients.

### Flow cytometry.

BM preparation and cell-surface staining were performed as described previously (16). In brief, BM cells were isolated by crushing long bones and hips, treated with red blood cell lysis buffer (150 mM NH_4_CL and 10 mM KHCO_3_), and washed with staining media (SM) (HBSS with % fetal bovine serum); 8 × 10^6^ unfractionated BM cells were stained for flow cytometry analysis. For sorting analysis, pools of unfractionated BM cells were purified by Ficoll separation (Histopaque-11191, MilliporeSigma 11191), then enriched for c-Kit^+^ cells using magnetic beads/autoMACS separation (Miltenyi Biotec). BM cells were stained with unconjugated rat lineage-specific antibodies (Ter119, Mac1, Gr-1, B220, CD5, CD3, CD4, CD8) or biotinylated lineage-specific antibodies (Gr-1, Mac-1, B220, Ter119, and CD3e), followed by staining with goat anti-rat PE-Cy5 secondary antibodies or streptavidin–eFluor 6450 secondary antibodies, respectively. Cells were then stained using c-Kit-APC-eFluor 780, Sca-1-PB, CD48-BV711 or CD48-PerCP-Cy5.5, CD150-PE or CD150-BV650, Flt3-biotin or Flt3-PE, CD62L-BV510, FcgR-BV510 or FcgR-PerCP-eFluor 710, CD34-FITC, EPCR-PerCP-eFluor 710, CD41-BV605, CD105-APC, and CD127-BV785 antibodies. Secondary staining was performed with streptavidin-BV711/ streptavidin-PE-Cy7. The Zombie NIR Fixable Viability Kit (BioLegend) was used for dead cell exclusion. For Hoechst 33342/Ki67 staining, surface-stained cells were fixed and permeabilized using a Cytofix/Cytoperm kit (BD) before staining with a PE-conjugated anti-Ki67 antibody and Hoechst 33342 (5 μg/ml). Flow cytometry reagents are presented in Supplemental Table 3. Cell sorting was performed on a FACSAria II (BD Biosciences). Analyses were performed on a 5 laser (16 ultraviolet [355 nm] channels, 16 violet [405 nm] channels, 14 blue [488 nm] channels, 10 yellow-green [561 nm] channels, 8 red [635 nm] channels) Aurora Spectral Flow Cytometer (Cytek Biosciences). Data analysis was performed using FlowJo (BD Biosciences) software.

### Tissue preparation for histology and flow cytometry.

Hearts were removed, fixed in 10% buffered formalin, treated on sucrose gradient, and embedded in OCT before being serially sectioned and stained with H&E dyes. Aortic valves were evaluated and graded by one investigator blinded to the study protocol. Prior to aorta harvest, hearts were perfused with 10 mL of PBS to limit blood contamination. Isolated aortas were mechanically dissociated before being incubated in an enzyme cocktail (450 U/mL collagenase type I, 125 U/mL collagenase type XI, 60 U/mL hyaluronidase type I-S, and 60 U/mL DNase I grade II) at 37°C for 50 minutes (62). Single-cell suspensions were filtered through a 70 μm filter and stained for flow cytometry analysis. For placenta studies, placentas were harvested from dams at gestation days 17–18 and mechanically dissociated for 1 to 2 minutes in 1 mL of cold StemPro Accutase Enzymatic Solution before incubation at 37°C for 35 minutes (63). After filtration through a 70 μm strainer and treatment with red blood cell lysis buffer, cells were washed and stained for flow cytometry.

### Confocal immunofluorescence microscopy.

From 500 to 1,000 HSCs were directly sorted on Polysine Microscope Slides (Thermo Fisher Scientific, P4981-001). Cells were allowed to adhere to the glass slide for 10 minutes, fixed using 4% paraformaldehyde (BD Cytofix, 554655) for 15 minutes, and permeabilized with 0.2% Triton X-100 (Sigma-Aldrich, T9284) for 20 minutes at room temperature. After blocking with 10% donkey serum (Sigma-Aldrich, D9663) for 20 minutes, slides were successively stained with anti-FOXO3 (EMD Millipore, 07-1719, dilution: 1/200) and Alexa Fluor 568–conjugated goat anti-rabbit (Thermo Fisher Scientific, A11011, dilution: 1/500) IgG (H+L) antibodies. After wash, slides were mounted using Gold Antifade Mounting Media with DAPI (Invitrogen, 536939) and analyzed with a LSM 710 confocal microscope system equipped with an inverted microscope (Observer Z1, Zeiss) using ×60 magnification at 1.2 AU pinhole 564 to give an optical section thickness of 0.39 μm images. Image analyses were performed using Imaris (Oxford Instrument, version 9.5) and NIS-elements (Nikon, version 5.20.02) software.

### BMDMs.

One million whole BM cells were plated on a 24-well plate in complete DMEM media (supplemented with 10% FBS, 1% penicillin/streptomycin in the presence of recombinant mouse macrophage colony-stimulating factor [M-CSF]; 20 ng/mL, BioLegend). After 6 days in culture, BMDMs were activated with recombinant murine IFN-γ, (20 ng/mL) and 20 ng/mL LPS (20 ng/mL) or media-only Ctrl. Cell numbers were determined 3 hours and 24 hours after activation using a hematocytometer and trypan blue for dead cell exclusion.

### Immunoblot analysis.

FACS-sorted LSK (5 × 10^4^) or AutoMACS-enriched c-Kit^+^ cells (2 × 10^5^) were used for immunoblotting analyses. Whole-cell lysates were prepared in 1× RIPA lysis buffer containing protease and phosphatase inhibitor cocktails. Cell lysates were resuspended in Laemmli buffer, boiled for denaturation, electrophoresed through 4%–15% SDS-PAGE gradient gel, and transfered to a PVDF membrane. Western blot analysis was performed with anti-DNMT1, anti-DNMT3A, and anti-ACTB antibodies and revealed with anti-mouse or anti-rabbit IgG secondary antibodies tagged with horseradish peroxidase (HRP).

### qRT-PCR analysis.

Quantitative reverse-transcription PCR (qRT-PCR) was performed using total RNA isolated from approximately 1 × 10^5^ cells. RNA was treated with DNase I and reverse transcribed using the SuperScript III Kit and random hexamers (Invitrogen). The cDNA equivalent of 200 cells per reaction was analyzed in triplicate on a 7900 HT Fast Real-Time PCR System (Applied Biosystems) using SYBR green reagents (Applied Biosystems) and gene-specific primers.

### DNA methylation analysis.

LSK cells were isolated by FACS, and DNA was extracted using the QIAamp DNA Mini Kit (QIAGEN) per the manufacturer’s instructions. Sequencing was performed at the CCHMC DNA Sequencing and Genotyping Core, with subsequent bioinformatics analyses performed by the CCHMC Bioinformatics Collaborative Services. Briefly, 60 ng of genomic DNA was bisulfite converted with the EZ DNA Methylation Kit (Zymo Research). Libraries were sequenced on the Illumina NovaSeq 6000 with 100 bp paired end reads and a depth of at least 30 million reads per sample. Reads were aligned to the mm10 reference genome by Bismark(version 0.18.2). CpG methylation calls were extracted from the alignment using the Bismark Methylation Extractor. MethylKit, version 1.12.0, in R, version 3.6.1, was used for methylation analysis. CpG sites covered in all samples of each group were considered for the downstream analysis. Methylation information was summarized over a tiling window of 1,000 bp in length across the whole genome. Differentially methylated regions (DMRs) with a percentage methylation difference larger than 0% and a *q* value of less than 0.25 were identified using the χ^2^ with overdispersion correction. DMRs were annotated using the genomation, version 1.18.0, and GenomicRanges, version 1.38.0, packages in R, version 3.6.1. DMRs in promoter, exonic, and intronic regions were subjected to Gene Set Enrichment Analysis (GSEA) using GSEA, version 3.0., for genes with multiple DMRs. The DMR with the highest percentage of methylation change was selected for GSEA analysis. Percentages of methylation change values for genes were used as a rank score to run the GSEAPreranked module in GSEA.

### ATAC-Seq analysis.

ATAC-Seq assays were performed as previously described on isolated nuclei from 50,000 sorted LSK cells (64). After the nuclei preparation, the transposase reaction was performed for 60 minutes at 37°C. The transposed DNA was purified using a QIAGEN MinElute Kit, and library fragments were amplified using 1× NEBNext PCR Master Mix. The libraries were purified with the SPRI Beads Double Size Selection (0.4/1.2×) (Beckman Coulter) and then sequenced on the Illumina NovaSeq X Plus with PE150, aiming at greater than 120M read pairs per sample. ATAC-Seq reads in the FASTQ format were subjected to quality control using FastQC, version 0.11.7, Trim Galore!, version 0.4.2, and cutadapt, version 1.9.1. The trimmed reads were aligned to the reference mouse genome version GRCh38/mm10 using Bowtie2, version 2.3.4.1, with parameters “--very-sensitive-local -X 2000”. Aligned reads were stripped of duplicate reads using sambamba, version 0.6.8. Peaks were called with MACS2, version 2.1.2, using the parameters “-g mm -p 0.01--shift -75 --extsize 150 --nomodel -B --SPMR --keep-dup all --call-summits”. Consensus peaks among all samples were obtained in 2 steps by selecting called peaks present in at least 75% of the biological replicates and by merging selected peaks at 50% overlap using BEDtools, version 2.27.0. The resulting sets of peaks were converted from BED format to a gene transfer format (GTF) to enable fast counting of reads under the peaks with the program featureCounts, version 1.6.2 (Rsubread package) (Bioconductor). Differential open chromatin regions (DOCs) between groups of samples were assessed with the R package DESeq2, version 1.26.0. Peaks were associated to nearest or overlapping gene and genomic features. GSEA was carried out using the GSEAPreranked script and hallmark gene sets.

### RNA-Seq analysis.

Ctrl, WT^STZ^, and Nlpr3^STZ^ placenta were dissected at G17 and placental CD45^+^ cells isolated by FACS sorting. Total RNA was purified using a RNeasy Micro Kit Column System (QIAGEN). RNA quality was controlled using an Agilent Bioanalyzer before processing for retrotranscription, linear amplification, and cDNA library generation. The whole transcriptome was amplified using the SMARTer Ultra Low RNA Kit for Illumina Sequencing (Clontech). cDNA libraries were prepared using Nextera XT DNA Sample Preparation Reagents. Fragmented and tagged libraries were pooled and were sequenced on an Illumina NovaSeq 6000 platform using a paired end 150 bp sequencing strategy. RNA-Seq reads in FASTQ format were subjected to quality control using FastQC, version 0.11.7, Trim Galore!, version 0.4.2, and cutadapt, version 1.9.1. The trimmed reads were aligned to the reference mouse genome, version mm10, with the program STAR, version 2.6.1e, and stripped of duplicate reads with the program sambamba, version 0.6.8 (5). Gene-level expression was assessed by counting features for each gene, as defined in the NCBI’s RefSeq database. Read counting was done with the program featureCounts, version 1.6.2, from the R subread package. Raw counts were normalized as transcripts per million (TPM). Differential gene expressions between groups of samples were assessed with the R package DESeq2, version 1.26.0. Gene list and log_2_ fold change were used for GSEA analysis using the Gene Ontology (GO) pathway data set.

### Multiplex cytokine analysis.

The concentration of cytokines was measured in duplicate in serum isolated from LPS-treated adult Ctrl and GD WT^STZ^ offspring using the Mouse High Sensitivity T-Cell 18-plex Discovery Assay (Eve Technologies, MDHSTC18).

### Statistics.

All results are expressed as means, with error bars reflecting SD. Statistical analyses were performed using GraphPad Prism, version 9. Ordinal variables (atherosclerosis grade) were analyzed using the χ^2^ test for trend. Differences between 2 groups were assessed using unpaired, 2-tailed Student’s *t* test. Data involving more than 2 groups were assessed by 1- or 2-way ANOVA with Tukey’s or Šidák’s post hoc test. *P* < 0.05 was considered significant.

### Study approval.

Mice were bred and housed in the AAALAC-accredited animal facility of CCHMC. All animal experiments were approved by the CCHMC IACUC.

### Data availability.

Next-generation sequencing data are available at the NCBI’s Gene Expression Omnibus database (GEO GSE244698). Values for all data points in graphs are reported in the Supporting Data Values file.

## Author contributions

VG performed and analyzed all the experiments with the help of SG, AA, and M Solomon. M Sakabe and NC performed all cardiac histopathology preparations, which were independently evaluated by AK. XZ and HLG provided expertise for ATAC-Seq analysis. AK and MX provided expertise and critical insights for the development of the project and manuscript. VG and DR designed, interpreted, and analyzed the studies and wrote the manuscript.

## Supplementary Material

Supplemental data

Unedited blot and gel images

Supplemental table 1

Supporting data values

## Version 1 11/21/2023

In-Press Preview

## Version 2 01/16/2024

Electronic publication
