# Supplementary material for: Gestational diabetes in mice induces hematopoietic memory that affects the long-term health of the offspring
Source: J Clin Invest. 2024 Jan 16;134(2):e169730. doi: 10.1172/JCI169730 (PMC10786695; doi:10.1172/JCI169730)
Supplement: Supplemental data [file jci-134-169730-s034.pdf]

SUPPLEMENTAL MATERIAL

Gestational diabetes in mice induces hematopoietic memory

that impacts the long-term health of the offspring

Vinothini Govindarajah, Masahide Sakabe, Samantha Good, Michael Solomon, Ashok Arasu, Nong Chen, Xuan Zhang, H. Leighton Grimes, Ady Kendler, Mei Xi and Damien Reynaud

- **Supplemental Figures 1-6**
- **Supplemental Tables 1-3**
  - Table S1: available as Excel File, provides differential gene expression / gene set enrichment analyses associated with the bulk RNAseq experiment performed on placental CD45+ cells (Figure 5G).
  - Table S2: List of key resources.
  - Table S3: List of Antibodies/reagents used for flow cytometry analysis.

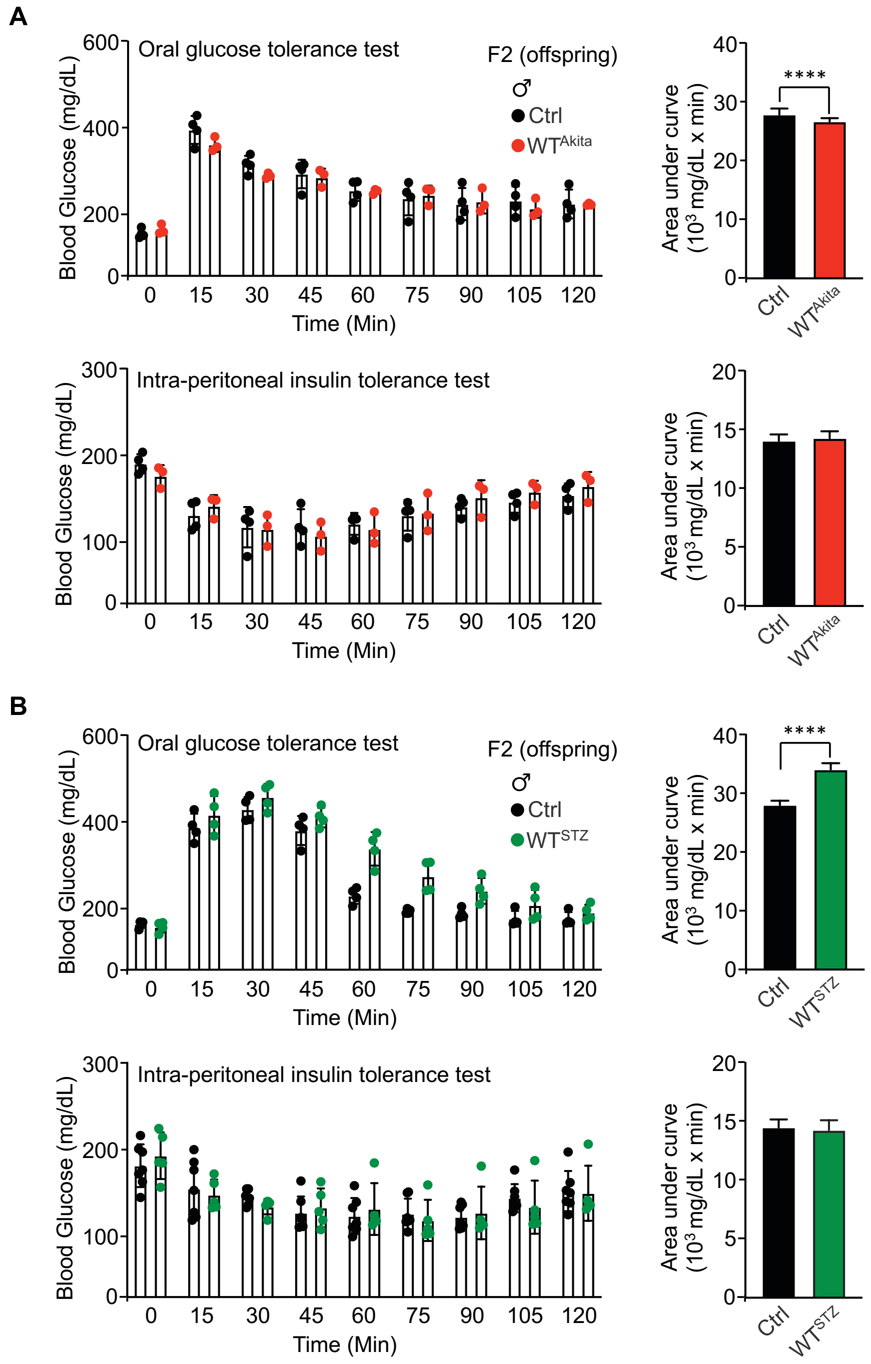

Supplemental Figure 1. Offspring born to diabetic pregnancy display minimal glucose control alterations. (**A-B**) Blood glucose levels of adult WT^{Akita} (A) and WT^{STZ} (B) offspring during oral glucose tolerance test [OGTT] (upper panel) and intra-peritoneal insulin tolerance test [IPTT] (lower panel) (A: n = 3-4/group; B: n = 4/group). Right graphs indicate difference in calculated area under the curve (AUC) compared to respective control. Graphs indicate mean ± SD. Unpaired Student's t test ****, $P \leq 0.0001$.

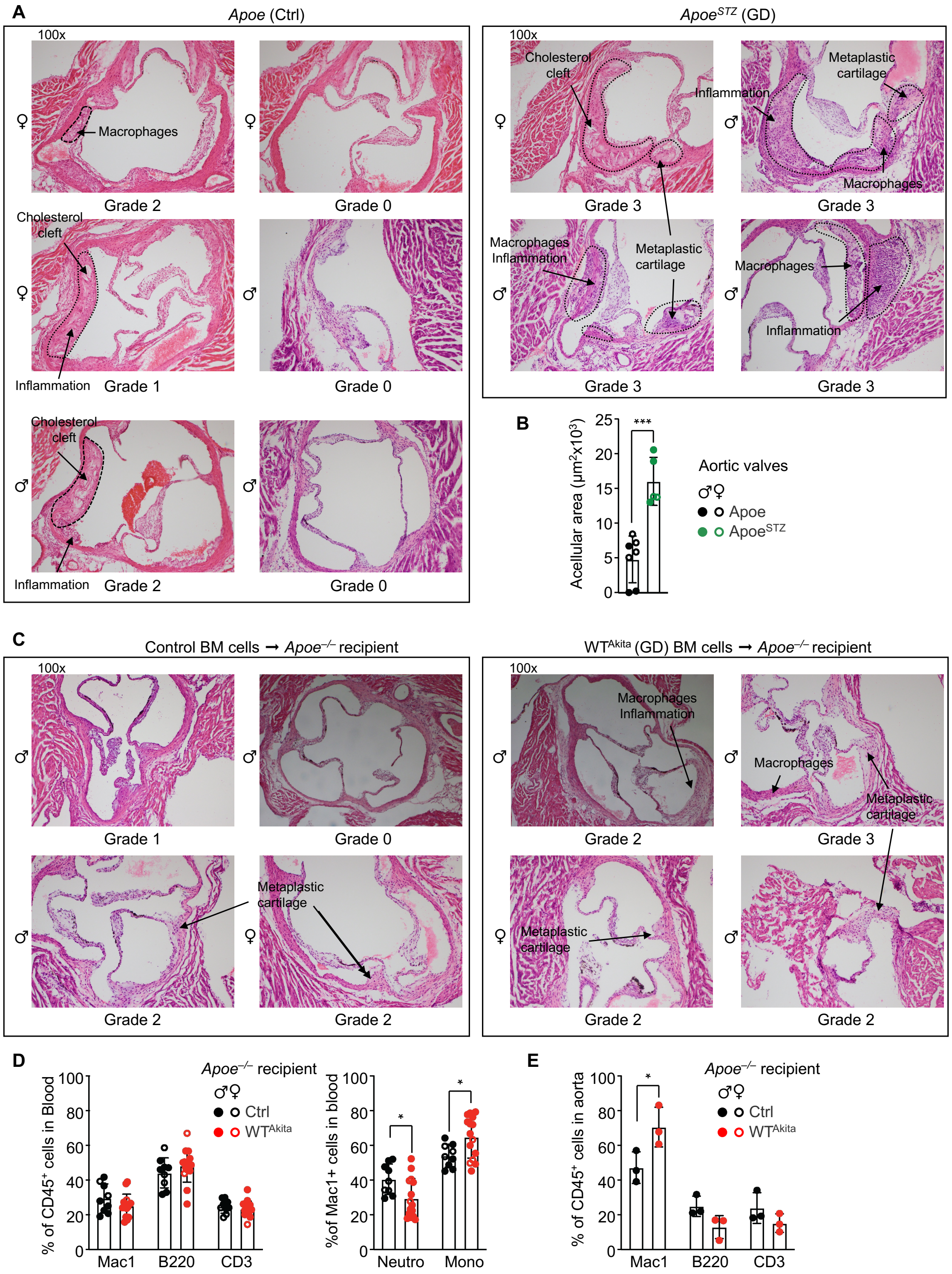

Supplemental Figure 2. GD is associated with aortic atherosclerosis severity in adult offspring. (**A**) Histological analysis of hematoxylin and eosin-stained aortic valves from *Apoe*$^{-/-}$ offspring born to diabetic pregnancy compared to control. 100x magnification (**B**) Acellular area identified in H&E stain lesions (n = 7-5/group). (**C**) Histological analysis of valves from *Apoe*$^{-/-}$ recipient mice transplanted with BM cells isolated from Ctrl or GD offspring. 100x magnification (**D**) Blood composition in *Apoe*$^{-/-}$ recipient mice transplanted with BM cells isolated from Ctrl or GD offspring. Left panel shows the percentage of myeloid (Mac1) and lymphoid (B220 and CD3) cells. Right panel shows the percentage of neutrophil (Ly6G^{+}) and Monocytes (Ly6G^{-} Ly6C$^{+/low}$) in myeloid cells (n = 9-15/group). (**E**) Blood cells in aorta isolated from *Apoe*$^{-/-}$ recipient mice transplanted with BM cells isolated from Ctrl or GD offspring (n = 3/group). Graphs indicate mean ± SD. Unpaired Student's t test (B) and two-way ANOVA with Sidak's post hoc test (D-E): *, P ≤ 0.01; ***, P ≤ 0.0005.

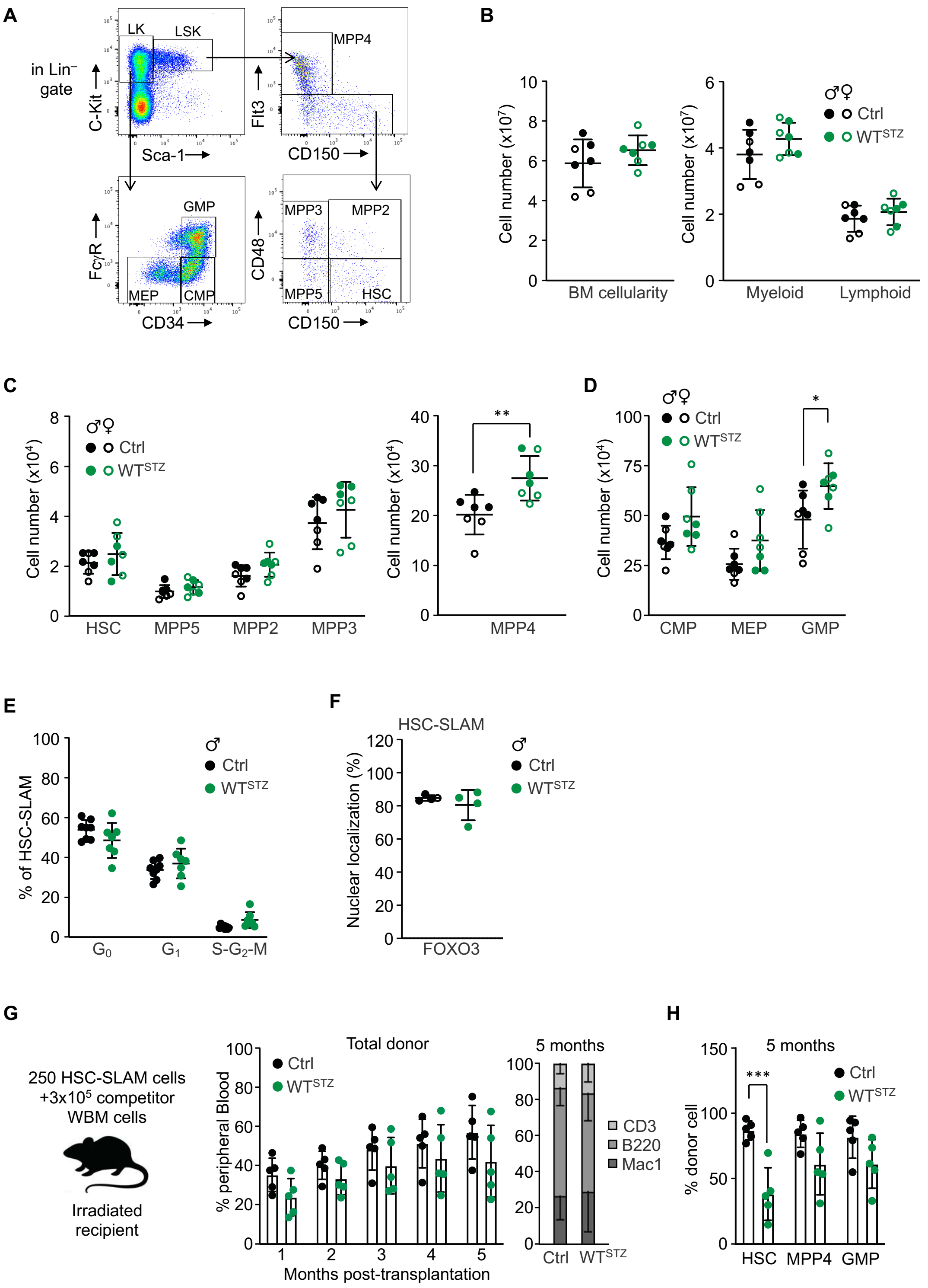

Supplemental Figure 3. Offspring born to diabetic pregnancy display altered steady-state hematopoiesis: (**A**) Representative FACS plot showing the phenotypic definition of BM HSPC compartments (**B**) BM cellularity and absolute number of BM myeloid/lymphoid cells in adult WT^{STZ} offspring (n = 7/group). (**C-D**) Absolute number of HSPC populations in the BM of adult Ctrl and WT^{STZ} offspring (n = 7/group). (**E**) Percentage of HSC distribution in cell cycle phases in adult Ctrl and WT^{STZ} offspring (n = 7-8/group). (**F**) Percentage of HSCs isolated from Ctrl and WT^{STZ} offspring that present FOXO3 nuclear localization at steady state (n = 4 with 50 individual cells analyzed for each). (**G-H**) Competitive hematopoietic reconstitution assay for HSCs isolated from Ctrl (n = 5) and WT^{STZ} (n = 5) offspring from 1 experiment: PB chimerism over time (G) and BM chimerism for HSPC compartments, 20 weeks after transplantation (H). Graphs indicate mean ± SD. Two-way ANOVA with Sidak's post hoc test (B, C D, G and H) or with Tukey's post hoc test (E and F): *, P ≤ 0.05, **, P ≤ 0.01; ***, P ≤ 0.0005.

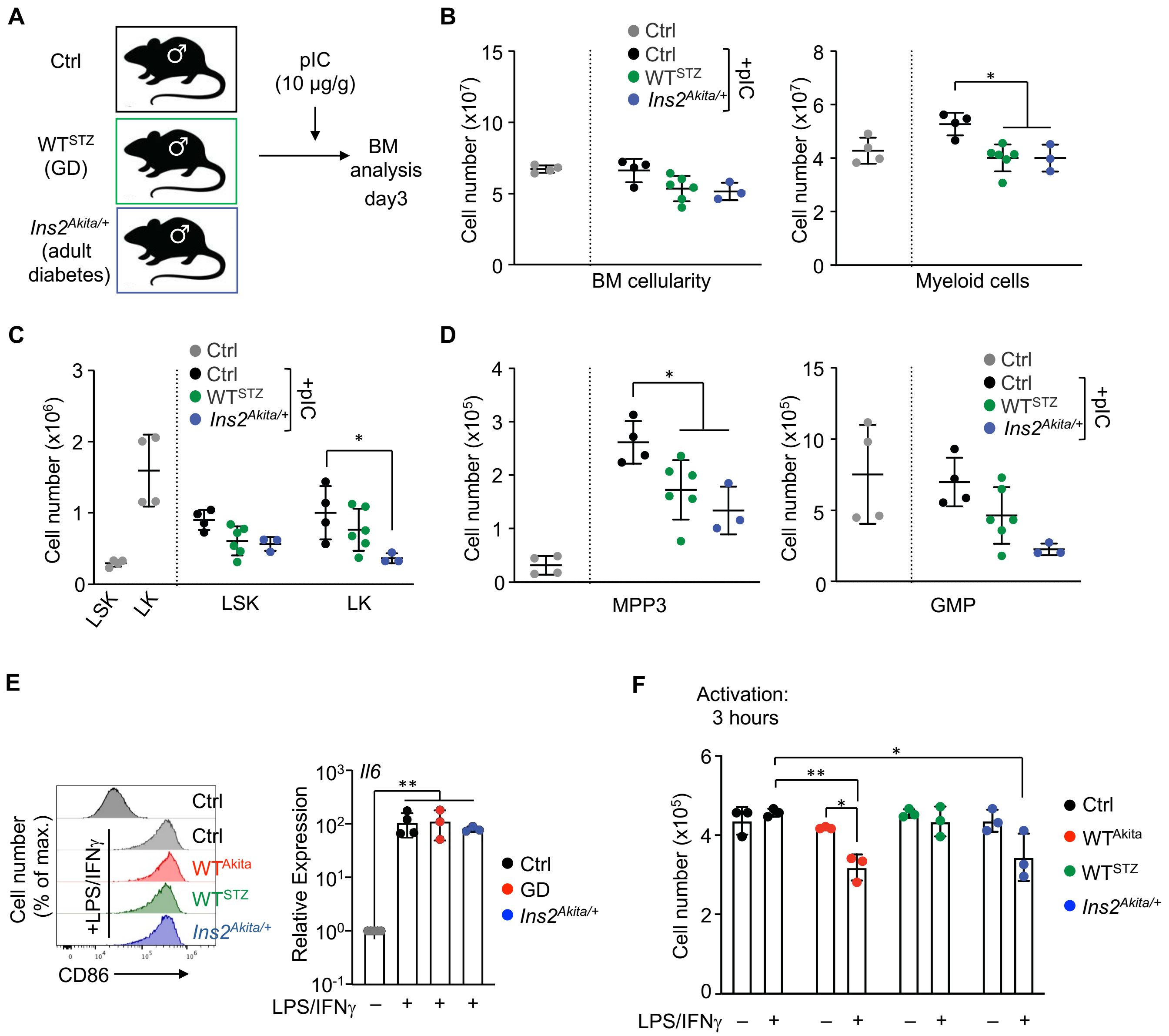

Supplemental Figure 4. Offspring born to diabetic pregnancy display altered in vivo and in vitro inflammatory response. (**A**) Schematic of the experimental design for the in vivo pIC inflammatory challenge (n = 4-6/group). (**B**) BM cellularity and absolute number of BM myeloid cell, 3 days after pIC treatment. (**C**) Absolute number of BM Lin$^-$Sca-1$^+$c-Kit$^+$ (LSK) and Lin$^-$Sca-1$^-$c-Kit$^+$ (LK) populations, 3 days after pIC treatment. (**D**) Absolute number of BM MPP3 and GMP population, 3 days after pIC treatment. (**E**) BMDM activated phenotype, 24 hours after LPS/IFNγ treatment. Left panel shows a representative FACS plots showing acquisition of the CD86 activation marker. Right panel show qRT-PCR analyses for *Il6* gene expression. Results are expressed as fold change relative to PBS-treated BMDMs, set at 1 (n = 3-4). (**F**) Absolute number of BMDM in culture 3 hours after with PBS or LPS/IFNγ (n = 3). Graphs indicate mean ± SD. One-way ANOVA with Tukey's post hoc test (B-D), two-way ANOVA with Sidak's post hoc test (E and F): *, $P \leq 0.05$, **, $P \leq 0.01$.

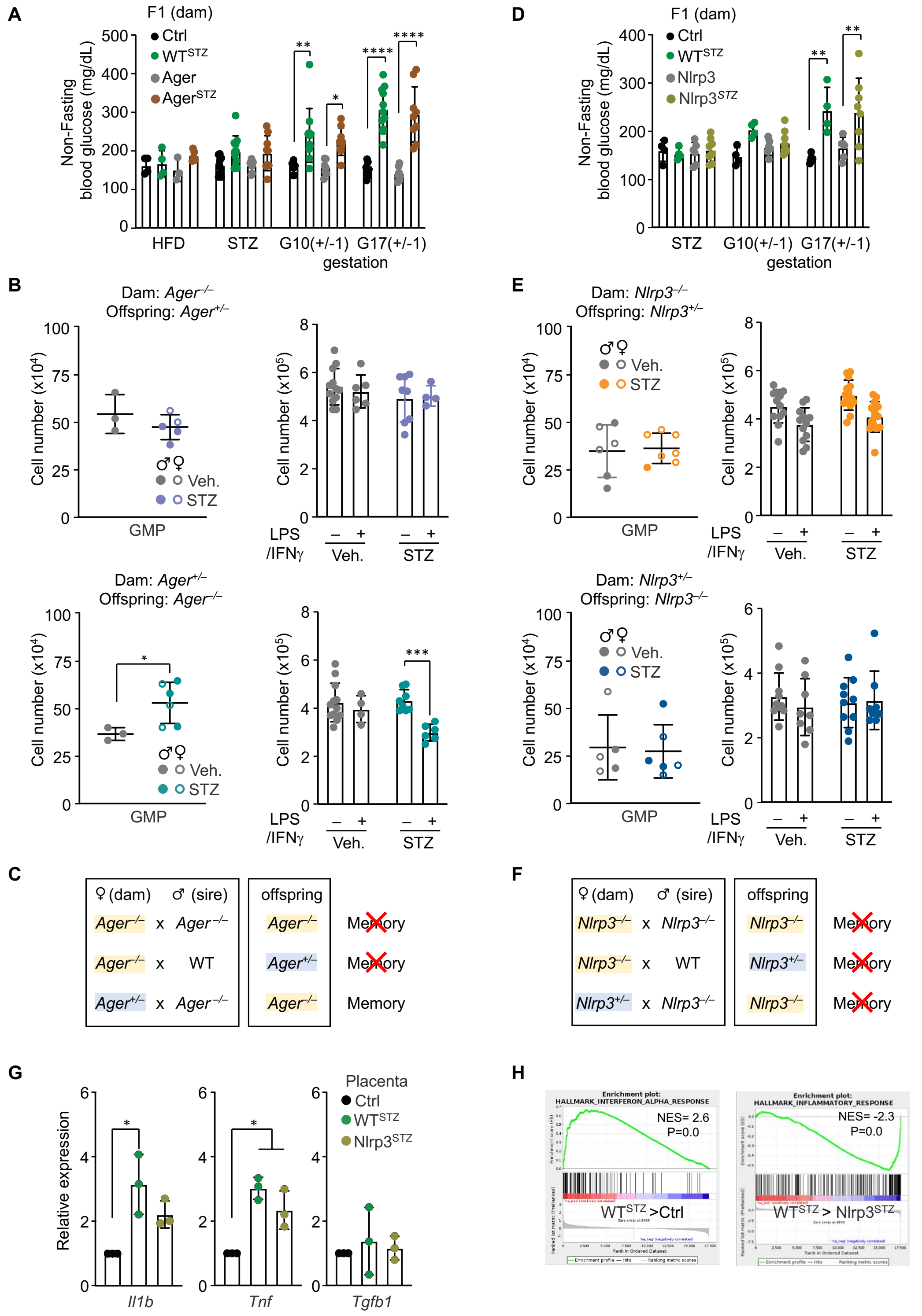

Supplemental Figure 5. Impact of maternal/fetal AGER and NLRP3 on the induction of GD hematopoietic memory. (**A**) Non-fasting glycemia in WT and *Ager*$^{-/-}$ pregnant dams following Vehicle or STZ treatment (n = 4-12/group). (**B**) Hematopoietic readout for offspring born to normal or diabetic pregnancy: absolute number of BM GMP cells (left graphs) and absolute number of BMDMs in culture 24 hours after treatment with PBS or LPS/IFNγ (right graphs) for *Ager*$^{+/-}$ offspring born to *Ager*$^{-/-}$ dams (upper panels) and *Ager*$^{-/-}$ offspring born to *Ager*$^{+/-}$ dams (lower panels) (n = 3-12/group). (**C**) Table summarizing the impact of maternal and fetal *Ager* knockout on the induction of the GD hematopoietic memory. (**D**) Non-fasting glycemia in WT and *Nlrp3*$^{-/-}$ pregnant dams following Vehicle or STZ treatment (n = 4-8/group). (**E**) Hematopoietic readout for offspring born to normal or diabetic pregnancy: absolute number of BM GMP cells (left graphs) and absolute number of BMDMs in culture 24 hours after treatment with PBS or LPS/IFNγ (right graphs) for *Nlrp3*$^{+/-}$ offspring born to *Nlrp3*$^{-/-}$

dams (upper panels) and *Nlrp3*$^{-/-}$ offspring born to *Nlrp3*$^{+/-}$ dams (lower panels) (n = 5-14/group). (**F**) Table summarizing the impact of maternal and fetal *Nlrp3* knockout on the induction of the GD hematopoietic memory. (**G**) RT-PCR analysis showing the expression of the *Il1b*, *Tnf* and *Tgfb*1 inflammatory cytokine genes. Results are expressed as fold change relative to controls, set at 1 (n = 3). (**H**) examples of gene signatures differentially enriched in placental CD45^{+} cells isolated from WT^{STZ} dams (compared to Ctrl and Nlrp3^{STZ} cells). Graphs indicate mean ± SD. One-way ANOVA with Tukey's post hoc test (G), two-way ANOVA with Tukey's post hoc test (A and D) or with Sidak's post hoc test (B and E, right graphs) and unpaired two-tailed Student's t-tests (B and E, left graphs): *, P ≤ 0.05, **, P ≤ 0.01, ***, P ≤ 0.0005, ****, P ≤ 0.0001.

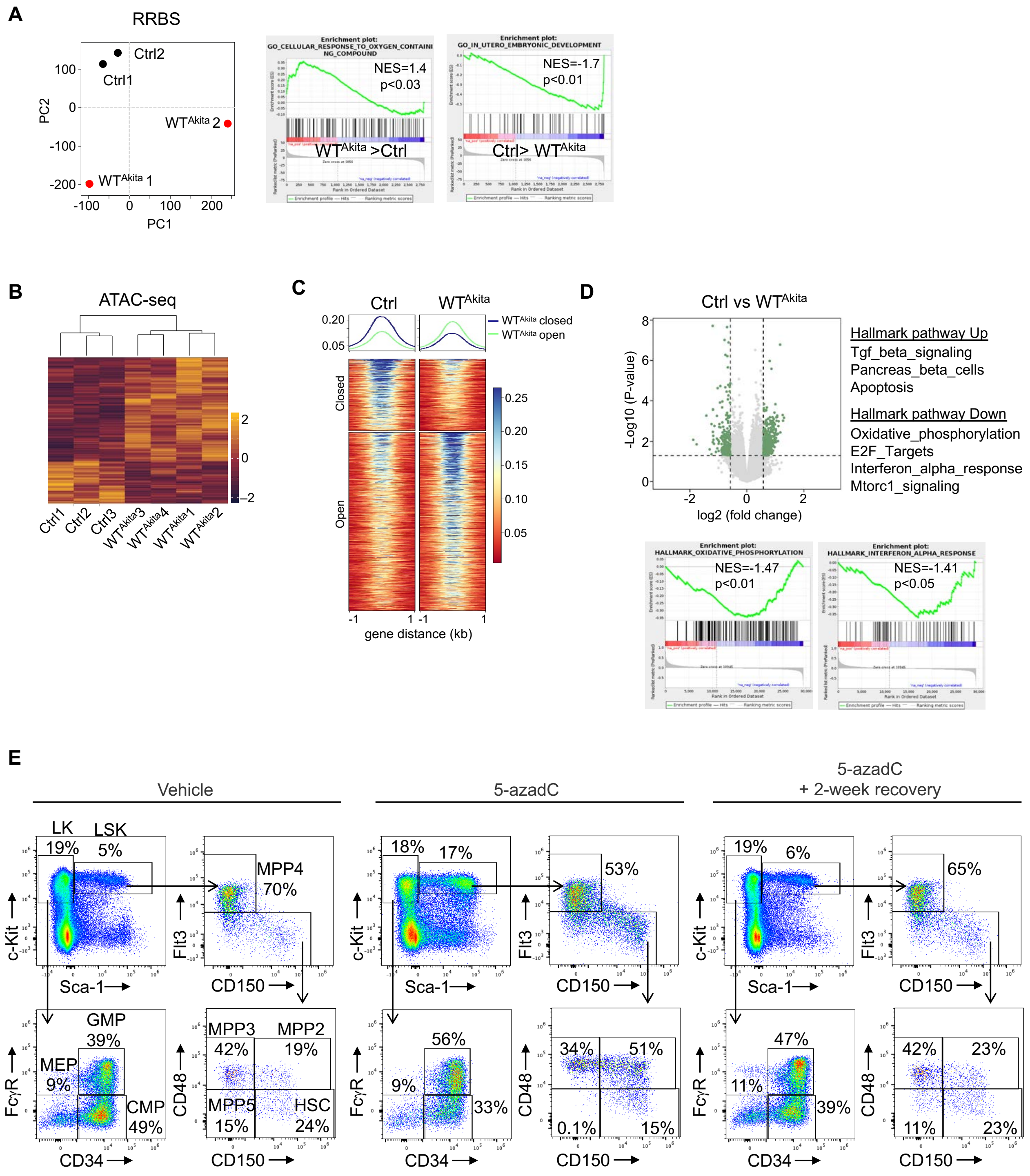

Supplemental Figure 6. Epigenetic alterations in LSK cells isolated from Ctrl and WT^{Akita} offspring. (**A**) DNA methylation profiling by reduced representation bisulfite sequencing (RRBS) in LSK cells isolated from Ctrl and WT^{Akita} offspring (n = 2). Right panel shows principal component analysis (PCA) visualization of the first two principal components of all differentially methylated genes. Left panel shows gene signatures differentially enriched in Ctrl and WT^{Akita} offspring. (**B**) Heatmap of read counts of differentially accessible chromatin regions with log2FC> |0.58| and P-value < 0.05 between Ctrl vs WT^{Akita} offspring (n = 3-4/group). (**C**) Density map for differentially accessible chromatin regions with log2FC> |0.58| and P-value < 0.05 between Ctrl vs WT^{Akita} offspring showing ±1kb around the ATAC-seq peak center. (**D**) Volcano plot of differentially accessible chromatin regions for Ctrl vs WT^{Akita} LSK cells. GSEA analysis and examples GSEA plot shows the negative enrichment of Oxidative Phosphorylation and Interferon alpha response pathways in WT^{Akita} LSK cells. (**E**) Representative FACS plots showing BM HSPC compartments in WT^{STZ} offspring treated with vehicle or 5-azadC, immediately after 4 weeks of treatment or 2 weeks of recovery (representative of 3).

Table S2. List of key resources

REAGENT or RESOURCE	Source	# Catalog
**Chemicals, peptides, and recombinant proteins**		
Recombinant mouse M-CSF	BioLegend	576402
Recombinant mouse IFNγ	BioLegend	752802
Lipopolysaccharide	Millipore Sigma	L2630
Polyinosinic–polycytidylic acid sodium salt	Millipore Sigma	P1530
D-glucose	ThermoFisher Scientific	A2494001
Streptozocin (STZ)	Millipore Sigma	S0130
5-aza-2′-deoxycytidine	Selleckchem	S1200
Gold Antifade mounting media /DAPI	ThermoFisher Scientific	536939
Cytofix/Cytoperm	BD biosciences	554655
donkey serum	Millipore Sigma	D9663
Foxo3 Antiboby	Millipore Sigma	07-1719
goat anti-rabbit Antiboby	ThermoFisher Scientific	A11011
Hoechst 33342	ThermoFisher Scientific	62249
DMEM media	ThermoFisher Scientific	10-016-CV
Fetal bovine Serum (FBS)	R&D Systems	S11550
penicillin/streptomycin	ThermoFisher Scientific	15140122
0.05% Trypsin-EDTA	ThermoFisher Scientific	25300-054
1X RIPA Lysis buffer	Cell Signaling Technologies	9806
protease inhibitor cocktail	Roche	04693159001
phosphatase inhibitor cocktail	Roche	046906837001
4× Laemmli sample buffer	Bio-Rad	1610747
DNMT1 Antiboby	Cell Signaling Technologies	5032
DNMT3A Antiboby	R&D System	MAB63151
β-ACTIN Antiboby	Millipore Sigma	A5441
anti–mouse IgG, HRP-linked Antibody	Cell Signaling Technologies	7076
anti–rabbit IgG, HRP-linked Antibody	Cell Signaling Technologies	7074
SuperScript™ III First-Strand Synthesis System	ThermoFisher Scientific	18080051
SYBR™ Select Master Mix	ThermoFisher Scientific	4472908
Control diet (13 Kcal% fat)	Lab Diets	5010
High fat diet (HFD) (60 Kcal% fat)	Research Diet	D12492
**Oligonucleotides**		
Il1b-Fw: tggcaactgttcctgaactca	IDT	N/A
Il1b-Rev: gggtccgtcaacttcaaagaac	IDT	N/A
Tnf-Fw: caaatggcctccctctcatca	IDT	N/A
Tnf-Rev: tgggctacaggcttgtcac	IDT	N/A

Il6-Fw: ccagaaaccgctatgaagttcc	IDT	N/A
Il6-Rev: gttgtcaccagcatcagtcc	IDT	N/A
Ccl2-Fw: agcagcaggtgtcccaaa	IDT	N/A
Ccl2-Rev: ttcttggggtcagcacagac	IDT	N/A
Tgfb1-Fw: gctgcgcttgcagagattaa	IDT	N/A
Tgfb1-Rev: gtaacgccaggaattgttgcta	IDT	N/A
Actb-Fw: ccctaaggccaaccgtgaaa	IDT	N/A
Actb-Rev: cagcctggatggctacgtac	IDT	N/A
**Software and algorithms**		
Prism 9	GraphPad	N/A
FlowJo	BD biosciences	N/A
Imaris and NIS 566 elements software	Oxford Instrument	N/A
Photoshop CC	Adobe	N/A
BioRender.com		N/A
**Experimental models: organisms/strains**		
C57BL/6J	The Jackson Laboratory	000664
B6.SJL-Ptprca Pepcb/BoyJ	The Jackson Laboratory	002014
Ins2$^{Akita/J}$	The Jackson Laboratory	003548
Apoe$^{-/-}$	The Jackson Laboratory	002052
Ager$^{-/-}$	The Jackson Laboratory	03277
Nlrp3$^{-/-}$	The Jackson Laboratory	02130
**Other**		
Polysine microscope slides	ThermoFisher Scientific	P4981-001
7900 HT Fast Real-Time PCR System	Applied Biosystems	N/A

Table S3. List of Antibodies/reagents used for flow cytometry analysis

Name	Other names	Clone	Fluorochrome	Source	# Catalog
Ter119	Ly-76	TER119	Purified rat	BioLegend	116202
Mac1	CD11b	M1/70	Purified rat	BioLegend	101202
Gr1	Ly-6C	RB6-8C5	Purified rat	BioLegend	108402
B220	CD45R	RA-3-6B2	Purified rat	BioLegend	103202
CD5		53-7.3	Purified rat	BioLegend	100602
CD3		17A2	Purified rat	BioLegend	100202
CD4		GK1.5	Purified rat	BioLegend	100402
CD8		53-6.7	Purified rat	BioLegend	100702
Ter119	Ly76	TER119	Biotin	BD biosciences	51-09082J
Mac1	CD11b	M1/70	Biotin	BD biosciences	51-01712J
Gr1	Ly-6C	RB6-8C5	Biotin	BD biosciences	51-01212J
B220	CD45R	RA-3-6B2	Biotin	BD biosciences	51-01122J
CD3	CD3e	17A2	Biotin	BD biosciences	51-01082J
Goat Anti-Rat		F(ab')2-IgG	PE-Cy5	ThermoFisher Scientific	A10691
Goat Anti-Rat		F(ab')2-IgG	Efl660	ThermoFisher Scientific	50-4017-82
Streptavidin			BV711	BioLegend	405241
Streptavidin			PE-Cy7	BioLegend	405206
Streptavidin			efluor450	ThermoFisher Scientific	48-4317-82
c-Kit	CD117	2B8	APC-eFluor780	ThermoFisher Scientific	47-1171-82
Sca-1	Ly-6a/e	D7	Pacific Blue	BioLegend	108120
Flt3	CD135	A2F10	Biotin	ThermoFisher Scientific	13-1351-85
Flt3	CD135	A2F10	PE	BioLegend	135305
CD48		HM48-1	BV711	BioLegend	103439
CD48		HM48-1	PerCP-Cy5.5	BioLegend	103422
CD48		HM48-1	Alexa-Fluor647	BioLegend	103416
CD150	Slamf1	TC15-12F12.2	PE	BioLegend	115904
CD150	Slamf1	TC15-12F12.2	BV650	BioLegend	115931
CD34	Mucosialin	RAM34	FITC	ThermoFisher Scientific	11-0341-85
FcγR	CD16/32	93	BV510	BioLegend	101333
FcγR	CD16/32	93	PerCP-eFluor710	BioLegend	46-0161-82
Ter119		Ter119	PE-Cy5	ThermoFisher Scientific	15-5921-82
Mac1	ITGAM, CR3, CD11b	M1/70	PE-Cy7	ThermoFisher Scientific	25-0112-81
Gr1	Ly6G/Ly6C	RB6-8C5	BV421	BioLegend	108433
CD3ε		1452C11	APC	BioLegend	100236
B220	CD45R	RA3-6B2	APC-eFluor780	ThermoFisher Scientific	47-0452-82
Ly6G		1A8	Pacific Blue	BioLegend	127611
Ly6C		HK1.4	PE	BioLegend	128007

CD45.1		A20	Alexa-Fluor700	BioLegend	110724
CD45.1		A20	BUV395	BD biosciences	565212
CD45.2		104	FITC	BioLegend	109806
CD45.2		104	BUV797	BD biosciences	612778
Ki67		16-A8	PE	BioLegend	652403
LIVE/DEAD			Zombie NIR	BioLegend	423105
UltraComp ebeads				ThermoFisher Scientific	01-2222-42
