# Supplementary figures and images for: Gestational diabetes in mice induces hematopoietic memory that affects the long-term health of the offspring
Source: J Clin Invest. 2024 Jan 16;134(2):e169730. doi: 10.1172/JCI169730 (PMC10786695; doi:10.1172/JCI169730)

unedited blot for Figure 6A

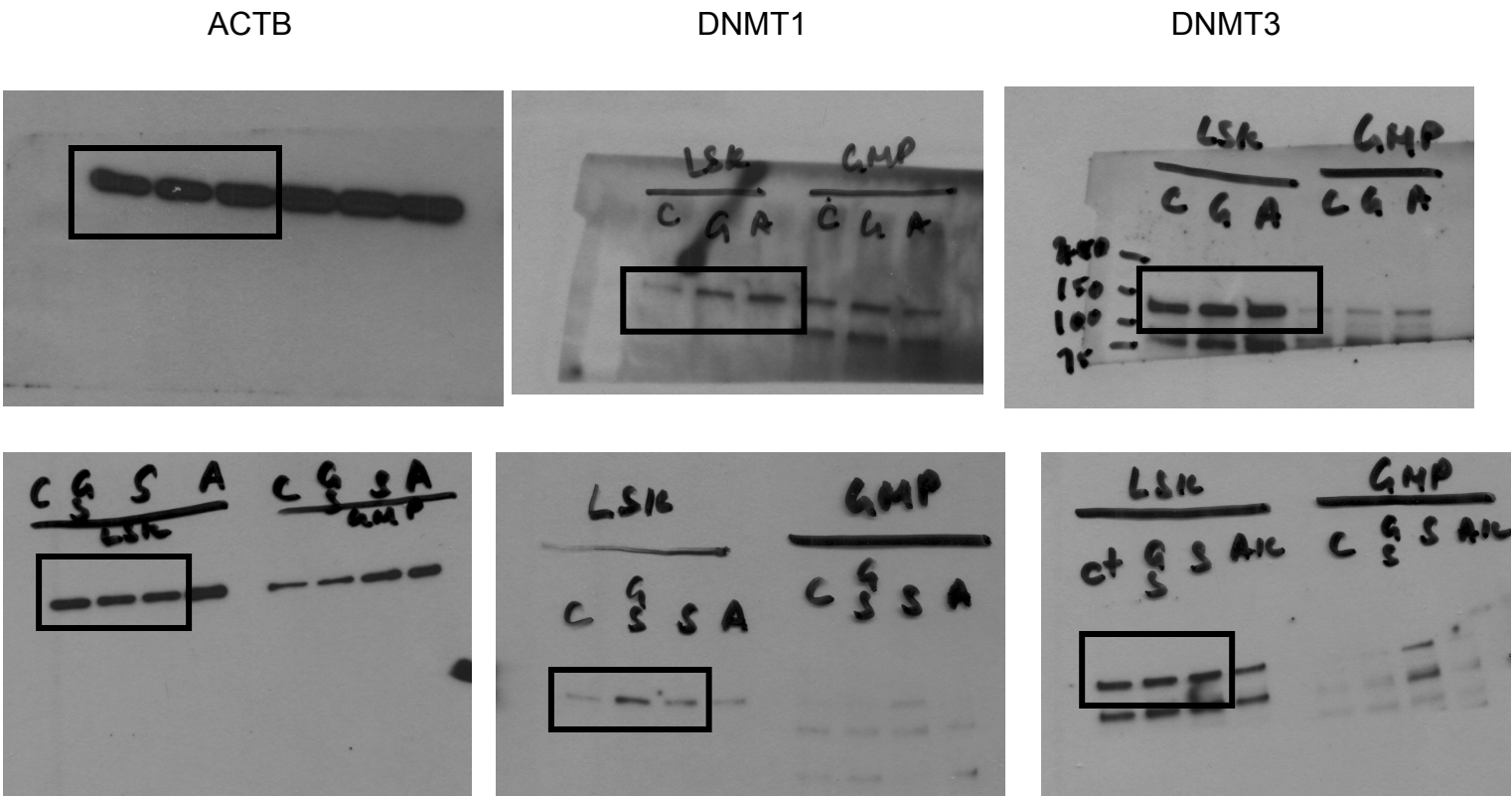

unedited blot for Figure 6D

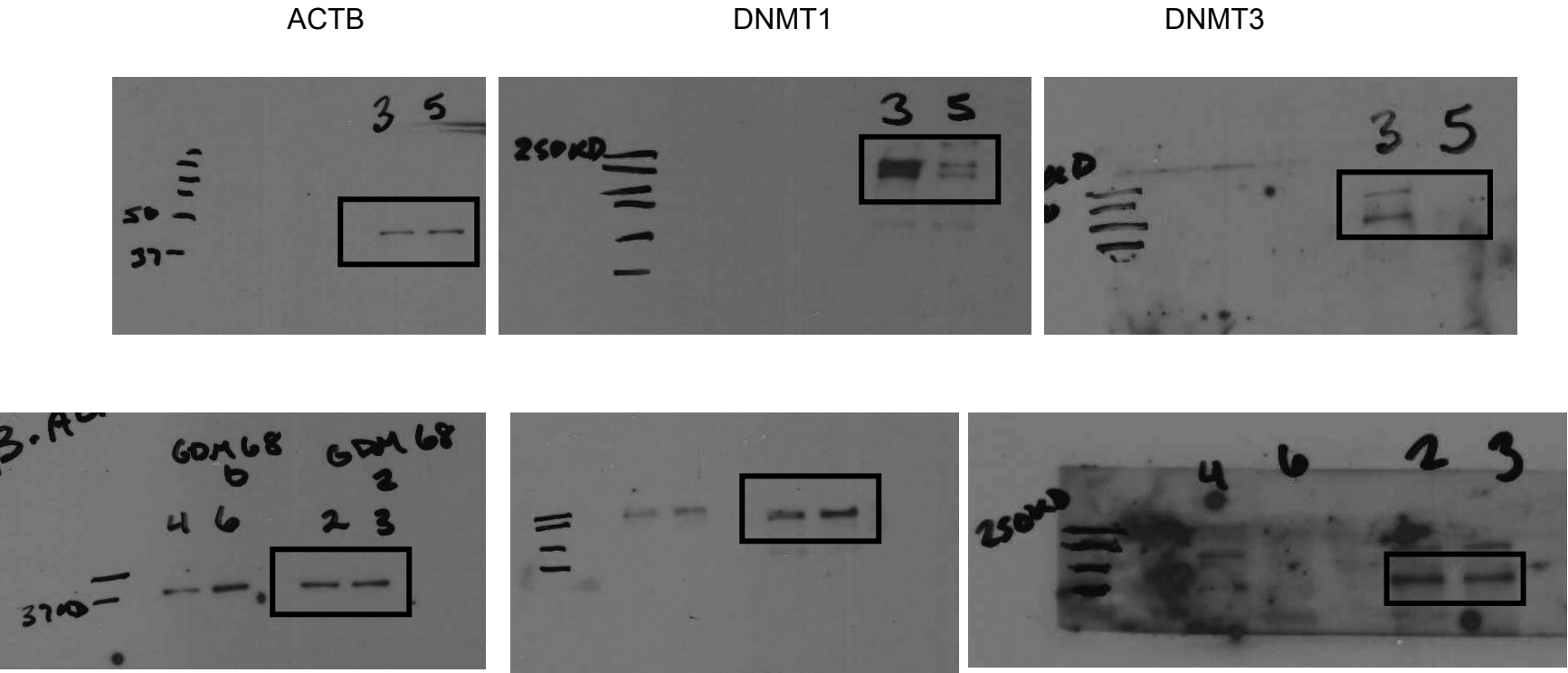

Supplement: Unedited blot and gel images [file jci-134-169730-s035.pdf]
